# Turning heterogeneity of statistical epistasis networks to an advantage

**DOI:** 10.1093/bib/bbaf699

**Published:** 2026-01-19

**Authors:** Diane Duroux, Federico Melograna, Héctor Climente-González, Bowen Fan, Andrew Walakira, Edoardo Efrem Gervasoni, Zuqi Li, Damian Roqueiro, Fabio Stella, Kristel Van Steen

**Affiliations:** BIO3—GIGA-R Molecular and Computation Biology, University of Liege, Place du 20 Août 7, B-4000 Liège, Belgium; BIO3—GIGA-R Molecular and Computation Biology, University of Liege, Place du 20 Août 7, B-4000 Liège, Belgium; BIO3—Department of Human Genetics, KU Leuven, Herestraat 49, B-3000 Leuven, Belgium; High-Dimensional Statistical Modeling Team, RIKEN Center for Advanced Intelligence Project, Chuo-ku, Tokyo 103-0027, Japan; Department of Biosystems Science and Engineering, ETH Zurich, Klingelbergstrasse 48, 4056 Basel, Switzerland; SIB, Swiss Institute of Bioinformatics, Switzerland, Amphipôle, Quartier UNIL-Sorge, 1015 Lausanne, Switzerland; Faculty of Medicine, Centre for Functional Genomics and Bio-Chips, Institute for Biochemistry and Molecular Genetics, University of Ljubljana, Vrazov trg 2, 1000 Ljubljana, Slovenia; Department of Informatics, Systems, and Communications, University of Milano-Bicocca, Viale Sarca 336, 20125 Milano (MI), Italy; BIO3—Department of Human Genetics, KU Leuven, Herestraat 49, B-3000 Leuven, Belgium; Department of Biosystems Science and Engineering, ETH Zurich, Klingelbergstrasse 48, 4056 Basel, Switzerland; SIB, Swiss Institute of Bioinformatics, Switzerland, Amphipôle, Quartier UNIL-Sorge, 1015 Lausanne, Switzerland; Department of Informatics, Systems, and Communications, University of Milano-Bicocca, Viale Sarca 336, 20125 Milano (MI), Italy; BIO3—GIGA-R Molecular and Computation Biology, University of Liege, Place du 20 Août 7, B-4000 Liège, Belgium; BIO3—Department of Human Genetics, KU Leuven, Herestraat 49, B-3000 Leuven, Belgium

**Keywords:** epistasis, GWAIS, IBD, heterogeneity, statistical epistasis networks

## Abstract

Epistasis detection is hindered by multiple challenges, including the proliferation of analytic tools and the diverse methodological choices made in Genome-Wide Association Interaction Studies (GWAIS). These factors often produce inconsistent and only partially overlapping results, with individual methods emphasizing distinct aspects of epistasis. Although comparative evaluations of GWAIS approaches exist, they generally do not identify the factors responsible for methodological discrepancies or assess their implications for biomedical research. Consequently, it remains unclear which features of GWAIS strategies contribute most to these differences and which methods are most appropriate for revealing specific genetic architectures. Here, we present a workflow designed to characterize heterogeneity in GWAIS results and derive practical recommendations systematically. First, we assess non-replicability by comparing single nucleotide polymorphisms-pair rankings and Statistical Epistasis Networks (SENs)—graphs in which nodes represent genetic loci and edges denote epistatic interactions—to identify clusters of protocols with similar outcomes. SENs provide a structured framework for visualizing and comparing variation in epistasis detection, enabling prioritization of interactions recurrently identified across methods. Second, we propose strategies to reduce heterogeneity and enhance robustness, with particular emphasis on interpretability. Notably, we demonstrate that differences among SENs can be informative rather than disadvantageous, as they yield complementary perspectives on disease genetics. Finally, we highlight the benefits of informed SEN aggregation, showing how this approach can strengthen the utility of GWAIS for elucidating biological mechanisms relevant to disease prevention, diagnosis, and management.

## Introduction

Epistasis studies are essential for understanding the genetic architecture of complex diseases. While Genome-Wide Association Studies (GWASs) have identified numerous causal variants, these variants often fail to fully explain underlying biological mechanisms. Genome-Wide Association Interaction Studies (GWAISs) complement GWASs by investigating epistasis, where the joint effects of genetic markers exceed their individual effects. GWAISs explore interactions between genetic loci that influence a phenotype beyond additive genetic effects. Epistasis has been frequently observed in model organisms [1, 2] and humans [3, 4].

The results of GWAISs can be represented as Statistical Epistasis Networks (SENs) [5], where nodes correspond to genetic loci [e.g. single nucleotide polymorphisms (SNPs) or genes], and edges indicate detected epistatic interactions. While SENs do not explicitly display phenotypic information, their edges reflect phenotype-associated interactions. SENs can be weighted based on interaction strength or binary when distinguishing significant interactions. These networks have facilitated discoveries in disease genetics, such as bladder cancer [5] and obesity, where an SEN of 709 SNPs and 1241 interactions helped identify key genes [6].

A multitude of computational methods have been developed to detect epistasis in GWAS data, vastly exceeding the number of tools available for single-locus detection [7]. Modeling higher order ($\geq 2$) genotype interactions is significantly more complex than single-SNP penetrance modeling, leading to diverse GWAIS methodologies. Since the advent of epistasis studies in GWAS, researchers have pursued various analytical strategies, each differing in interpretation, computational approach, and biological applicability [8].

Heterogeneity in GWAIS protocols often translates into variability at the results level, particularly in SENs, with implications for clinical applications. SEN-based analyses can uncover higher order interactions and pathways relevant to disease etiology, impacting prevention and diagnosis. Integrating GWAIS networks with external gene–gene interaction networks, such as protein–protein interaction networks, may reveal key molecular mechanisms. However, variability across GWAIS outputs necessitates an understanding of whether discrepancies reflect methodological limitations or meaningful biological insights.

Traditional GWAIS comparisons on synthetic data often fail to capture real-world biological complexity. A method’s theoretical advantages may not translate effectively to non-synthetic datasets due to limitations in modeling complex interactomes. To address this, we propose a workflow to assess the heterogeneity of SENs—or more generally, ranked lists of GWAIS outputs—by applying multiple GWAIS protocols to inflammatory bowel disease (IBD) GWAS data [9]. We use ranking and network comparison methods to identify clusters of tools yielding similar results. Potential sources of heterogeneity considered include search space size, population structure adjustment, epistasis significance assessment, and SNP-to-gene mapping strategies, though additional sources can be incorporated. Our workflow is validated on real-world data to assess its effectiveness in improving the robustness of epistasis findings.

This paper is structured as follows: Section 2, along with its Supplementary Section, details the study materials and data. Section 3 presents and discusses the results of the proposed workflow in the context of IBD, focusing on how it helps identify and mitigate heterogeneity across different GWAIS protocols. Section 4 provides a broader discussion of the workflow’s implications for translational epistasis research.

## Materials and methods

For the first time, we applied network theory to cluster epistasis results, enabling more robust and interpretable findings. To achieve this, we utilized NetANOVA [10], a newly proposed, context-appropriate tool that not only forms clusters but also determines the optimal number of clusters autonomously. This clustering method is fully unsupervised, assesses statistical significance internally, and is particularly suited to the current context, as will be shown later.

### Input data and preprocessing

In this project, we analyzed data on IBD, which includes ulcerative colitis (UC) and Crohn’s disease (CD) as its two main categories, along with other noninfectious bowel inflammations[11]. Studies reported a complex IBD genetic component with positive family history being a high-risk factor [12]. GWAS have detected more than 200 IBD-associated loci [13], but they only partially explain IBD genetic variance [14]. Epistasis could participate in understanding this missing heritability [15, 16]. We investigated the IBD dataset from the International Inflammatory Bowel Disease Genetics Consortium (IIBDGC). This dataset was genotyped on the Immunochip SNP array [17]. We performed quality control as in Ellinghaus *et al*. [9]. It reduces the number of SNPs from 196 524 to 130 071. The final dataset contained 66 280 samples, including 32 622 cases and 33 658 controls.

Since the IIBDGC dataset aggregates multiple cohorts, confounding of GWAIS results by shared genetic ancestry is a concern. As in Ellinghaus *et al*. [9], we used the first seven principal components to model population stratification. We adjusted the phenotypes for epistasis detection methods that cannot include covariates. We derived these corrected phenotypes by regressing out the principal components in a logistic regression model and subtracting model-fitted values from observed phenotype values (i.e. response residuals). In this work, we evaluate the impact of using binary phenotypes (with or without PCs as covariates) or the continuous (corrected) phenotypes.

Furthermore, data were quality controled as in Duroux *et al*. [18]. We removed rare variants (MAF < 5%) or in Hardy–Weinberg equilibrium ($P$-value < .001). All risk SNPs described in Liu *et al*. [19] were included, despite not meeting the previous quality control criteria. Moreover, if the two epistatic SNPs of a pair were located in the HLA region (chromosome 6 from Mb 25 to Mb 34), we rejected the pair because it is complex to differentiate between main and epistasis effects in the HLA region [20]. Finally, we discarded SNP pairs where the SNPs were in high linkage disequilibrium (LD $r^{2}~>$ 0.75), as recommended in Gusareva and Van Steen [21].

Computational limitations associated with some GWAIS methods required to take additional steps. We used ReliefF [22] on the processed data for SNP prioritization. Relief-based algorithms are feature selection methodologies that seek a balance between computational efficiency and sensitivity to complex patterns of association, such as interactions. The output is a ranked set of SNPs that one can use for feature selection. We also applied epiScan [23] on the processed data to reduce the search space via a fast exhaustive pairwise epistasis scan. We computed the top 10 000 SNP pairs and used the associated SNPs as input to other epistasis detection tools.

As GWAIS interpretation strategies often lie at the gene level, we compared two strategies to map SNP pairs to gene pairs. We call them *positional* and *functional* [18]. In the *positional* strategy, we searched for epistasis between SNPs that passed quality controls. We mapped the significant SNP interactions to gene interactions using a positional mapping obtained from FUMA’s SNP2GENE function [24]. We mapped an SNP to a gene when the genomic coordinates of the former were within the boundaries of the latter $\pm $ 10 kb. The *positional* dataset contains 38 225 SNPs. In the *functional* strategy, we used FUMA’s eQTL mapping that is based on eQTLs obtained from GTEx [25]. We mapped an eQTL SNP to its target gene when the association $P$-value was significant in any tissue (FDR $< 0.05$). The *functional* mapping includes a second step: we only considered the resulting gene pairs with prior evidence for interaction in Biofilter [26]. Biofilter contains pairs of genes with evidence of co-function across multiple publicly available biological databases. Given this set of candidate gene pairs and the eQTL mapping, we discarded all the SNPs that did not participate in any corresponding SNP pairs. The *functional* dataset contains 16 652 SNPs. Hence, the *positional* and the *functional* filters use different degrees of biological knowledge. Importantly, this is general biological knowledge and not information specific to IBD. Additionally, we removed self-interactions for both strategies because detecting within-gene epistasis requires special considerations. We applied epistasis tools to these two sets and compared the detected interactions.

### Modeling: epistasis detection tools

We considered 10 epistasis detection algorithms (see Table 1): linear regression [27], MB-MDR [28], (PLINK’s) BOOST [29], lightGBM [30], Epi-GTBN [31], Epiblaster [32], Neural Network Weights [33], Bayesian model [34], AntEpiSeeker [35], and CASMAP [36]. A brief description of each method and details about how they were used in this paper are given as Supplement section 1. All analyses were performed on a cluster running Scientific Linux release 7.2 (Nitrogen).

**Table 1:** Highlighted properties of the different epistasis detection algorithms and associated input types. Linear regression and BOOST refer to their PLINK implementations. The fifth column indicates whether the epistasis analysis was performed on binary phenotypes (cases versus controls) or phenotypes adjusted for population structure (continuous). The sixth column indicates whether missing SNP values were imputed before applying the epistasis detection tool

Method	Category	Test conditional	Covariates	Continuous	Genetic
		on main effects	adjustment	phenotypes	imputation
AntEpiSeeker [35]	Statistic	No	No	No	Yes
Bayesian model [34]	Network, Statistic	Yes	No	Yes	Yes
(PLINK’s) BOOST [29]	Statistic	Yes	No	No	No
CASMAP [36]	Statistic	No	Yes	No	Yes
Epiblaster [32]	Statistic	Yes	No	Yes	Yes
Epi-GTBN [31]	Network	No	No	No	Yes
LightGBM [30]	Machine learning	No	No	Yes	No
Linear Regression [27]	Statistic	Yes	No	Yes	No
MB-MDR [28]	Dimensionality reduction	Yes	Yes	Yes	No
Neural Network Weights [33]	Network, ML, statistics	Yes	No	Yes	Yes

### Inferential reproducibility and interpretation

#### Comparative analysis of epistasis outputs

The comparative analysis of epistasis outputs can be performed both at the SNP level and, to facilitate biological interpretation, on the gene level. We refer to Supplementary Section 3 for the analysis at the SNP level.

Here, we focused on gene-based SENs rather than SNP-based SENs. This involved converting the SNP-level results into gene-level results [18]. To obtain candidate gene pairs, we used the filters and mapping described in Section 2.1. Multiple strategies were applied to define which interactions to consider in the SENs. For protocols outputting $P$-values, we set the gene-pair $P$-values to $p_{G_{i},G_{j}}=min(p_{S,S})$, with $p_{S,S}$ the set of $P$-values of SNP interactions that are mapped to the gene pair $G_{i},G_{j}$. To minimize false positives at the gene level with AntepiSeeker, we applied the procedure suggested by the authors [35], which consists of first removing SNP pairs containing a SNP present in other pairs with a lower $P$-value (see Supplementary Section 1 ). Notably, since this procedure discarded most detected interactions, we did not apply this stringent filter in the analysis at the SNP level (Supplementary Section 3). If the outputted $P$-values were already adjusted for multiple testing (MB-MDR, CASMAP, antEpiSeeker), the significance threshold was 0.05. For tools providing $P$-values without correction for multiple testing we derived two theoretical thresholds. For the *positional* SNP-to-gene mapping scenario


\begin{align*} &t_{positional}=\frac{0.05}{\binom{\#G \in PositionalMap}{2}} = \frac{0.05}{117\,864\,981}=4.2\times10^{-10},\end{align*}

with $\#G \in PositionalMap$ the number of distinct genes in the set of *positional* SNP-to-gene mappings. For the *functional* mapping scenario


\begin{align*} &t_{functional}=\frac{0.05}{\#(G_i,G_j)\in U}=\frac{0.05}{18\,763}=2.6\times10^{-6}, \end{align*}

with U={$G_{i}, G_{j} \in $*functionalMap* & $(G_{i},G_{j}) \in $ Biofilter}. Hence, $\#(G_{i},G_{j})\in U$ is the number of pairs of genes where each gene is present in the set of *functional* SNP-to-gene mappings, and the pair of genes is also present in Biofilter gene-interactions. These theoretical thresholds were applied to the linear regression, BOOST, and epiblaster outcomes. For the linear regression, we also included the SENs derived in Duroux *et al*. [18] where the significance SNP-pair thresholds were calculated experimentally using 400 permutations ($t_{Positional}=1.1\times 10^{-10}$ and $t_{Functional}=6.2\times 10^{-8}$). The gene-pair significance assessment was derived from the Adaptive Truncated Product Methodology [37]. We called these SENs *linear regression Duroux*. Then, we set the following thresholds for tools, which only indicate the association strength. In epi-GTBN, 10 runs were performed with perturbed data, and the outcome contained the number of times each relation between two SNP appeared in the 10 runs. A pair was considered significant if it appeared in at least two runs. We set the weight threshold in the Neural Network Weights algorithm to $0.7\times max(obserserved_{weight})$. In other words, we selected interactions based on a comparison with the most significant ones. The same approach was applied for lightGBM outcome (*mean*). Since the lower the *mean*, the stronger the association, we first computed the association strength as $strength=\frac{1}{1+mean}$. Then, we fixed the threshold as $0.7\times max(observed_{strength})$. These two thresholds resulted in gene pair sets for SEN construction comparable with those generated by the other protocols. The SENs obtained from each epistasis detection protocol were then binarized; an edge was deemed present if it passed the threshold and was absent otherwise.

To identify which protocol properties produced close results, we clustered SENs into homogeneous groups using netANOVA [10]. NetANOVA assesses statistical significance between clusters of graphs. We refer to Supplementary Section 4 for an in-depth description of netANOVA.

#### Aggregation of homogeneous SENs

NetANOVA generates clusters of homogeneous graphs. To characterize the properties of each cluster, we subsequently synthesized the information within clusters. In other words, we aggregated multiple networks of a cluster into a single representative network. This process can be seen as link prediction in a representative graph. Link prediction [38] usually predicts edges (binary or weighted) in a network. Here, it is used to predict edges in a summary network from multiple binary graphs of a homogeneous cluster. It could be performed with simple algorithms such as the intersection (the edge is present in the summary network if it is present in all the cluster networks), the union, or the majority vote. More advanced aggregation methods have been discussed [39], and Latent Class Analysis (LCA) has been identified as relevant to merging and summarizing network information. Hence, we used the R [40] function *poLCA* [41], which allows a dataset to be partitioned into exclusive clusters called *latent classes*. Details on *poLCA* and a rationale of LCA choice are shown in Supplementary Sections 5 and 6.

#### Simulation data

Simulation data were generated using the EpiGEN framework [42] to model genetic associations and epistatic interactions within a synthetic African American population. The simulations produced genotype and phenotype datasets that mimic realistic patterns of genetic variation and disease risk, incorporating both marginal and interaction effects among predefined SNPs. Simulations included $10\,000$ individuals and $1000$ SNPs, with minor allele frequencies for disease-associated variants ranging between $0.1$ and $0.3$. A low effect-size setting was used ($\alpha = 5$ for marginal effects and $\alpha = 3$ for epistasic effects). The underlying model simulated different types of genetic effects. Marginal effects were assigned to two independent SNPs, while several distinct epistatic interaction patterns were modeled: joint-dominant, exponential, multiplicative, joint-recessive, and a higher order triplet interaction. In total, 10 SNPs were designated as epistatic loci, participating in pairwise and higher-order interaction models.

These configurations introduced complex, nonadditive relationships between alleles, representing realistic gene–gene interaction scenarios. The genotypes were encoded numerically ($0 =$ homozygous reference, $1 =$ heterozygous, $2 =$ homozygous alternate) for each SNP across all individuals, while the phenotypes were generated as binary traits. The simulation outputs also included information on the causal SNPs, their positions, and corresponding allele frequencies, allowing precise mapping between model design and observed outcomes. The resulting dataset represents a controlled genetic landscape where the effects of both individual SNPs and multi-locus interactions on disease risk can be studied systematically.

## Results

### Spotting the variance: detecting heterogeneity

**Figure 1:**
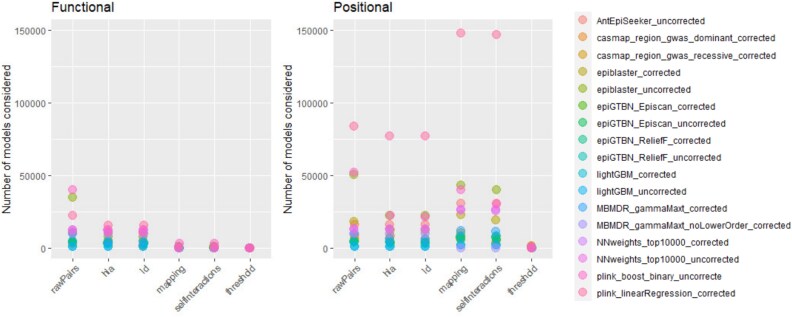
Characteristics of the 34 GWAIS protocols. The number of epistatic models resulting from successive quality controls, gene mappings, and significance filters is shown on the left for the *functional* protocols and on the right for the *positional* protocols. *RawPairs* gives the number of SNP pairs retained in the output of each epistasis detection tool. *HLA* provides the number of SNP pairs after removing pairs with the two SNP in the HLA region. *LD* corresponds to the number of SNP pairs that are not in high linkage disequilibrium ($r^{2}<0.75$, see Section 2). *Mapping* gives the number of quadruple SNP pairs–gene pairs obtained from the mapping strategies. *Self-interaction* reports the number of quadruples containing two different genes. *Threshold* is the final number of gene pairs that pass the significance cutoff (Section 2.3.1).

Implementing the selected GWAIS protocols (section 2.1) yielded 34 epistasis outputs at the SNP level. Two of these protocols directly generated genetic interaction results at the gene level by aggregating genetic information within each gene and examining interactions between these gene summaries (Bayesian model). It is important to note that this approach differs from strategies that convert or aggregate SNP–SNP interaction findings into gene–gene interaction evidence after conducting SNP-level GWAIS [18]. Figure 1 shows the size of the GWAIS protocols outputs in the successive quality controls, SNP-to-gene mapping, and significance cutoff steps. In the *functional* protocols, the two steps reducing the most the number of models are the HLA filter, where we removed pairs if the two SNPs are in the HLA region (chromosome 6 from Mb 25 to Mb 34) and the SNP-pair to gene-pair mapping. The latter was expected since the mapping involved a stringent gene-pair filtration based on Biofilter 37 266 candidate interactions. In the *positional* protocols, linear regression contained more pairs than the other protocol in most steps because we allowed uncorrected $P$-values up to.0001 to be kept in the output. Still, after the significance assessment, the number of gene pairs considered is in the range of the other tools. The *functional* protocols gave rise to fewer interactions with on average 14 significant gene pairs, compared with *positional* protocols containing on average 351 significant gene pairs. This difference mainly came from the difference in considered SNPs (38 225 versus 16 652 SNPs) and the reduction to Biofilter candidate gene pairs in the *functional* protocols.

**Figure 2:**
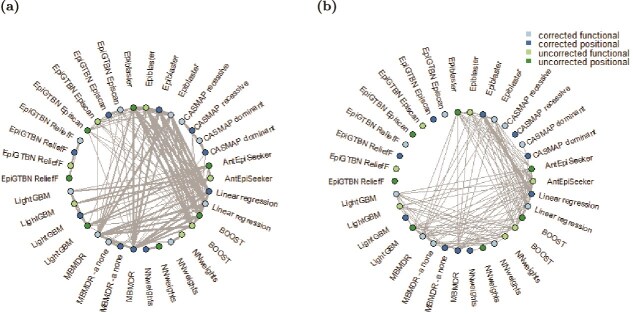
(a) Co-occurrence network based on top 1000 SNP-pair lists for considered epistasis detection protocols. (b) Resemblance network based on similarity between ranked output lists for top 1000 SNP-pairs. For readability, the top 20% most similar pairs of approaches are shown. Colors indicate whether epistasis detection involved phenotype correction for population structure and whether SNP set reduction relied on biological knowledge *functional* (or not: *positional*).

We first conducted a co-occurrence analysis of the obtained GWAIS results by calculating the number of shared epistatic pairs among the top 1000 interactions in each pair of protocols (Supplementary Section 3) (Fig. 2a). After HLA and LD quality controls, only lightGBM *positional* produced less than 1000 pairs (985 pairs). In the co-occurrence network, the wider the edge between two protocols, the more SNP pairs the protocols share in common in their top 1000 SNP pair lists. Epiblaster, BOOST, and linear regression had the highest number of similar top pairs. The *positional* linear regression and Epiblaster outcomes shared 944 pairs, and the corresponding *functional* outcomes had 925 SNP interactions in common. Then, *positional* BOOST and Epiblaster shared 530 pairs, and the corresponding *functional* outcomes had 525 similar interactions. To a lesser extent, MB-MDR was also close to linear regression (317 pairs) and BOOST (328 pairs) in the *positional* SNP-to-gene mapping scenario. In addition, there were high similarities between protocols based on the same analytic tools and different input settings, i.e. *positional* versus *functional*: antEpiSeeker (593 pairs), Epiblaster (472 pairs), BOOST (462 pairs), MB-MDR without lower-order effect correction (405 pairs), and CASMAP dominant (361 pairs). This co-occurrence analysis also showed that protocols using the same tool and input data with phenotypes either corrected or uncorrected for population structure led to close results in the top 1000 SNP pairs: Neural Network Weights *functional* (405 pairs), Neural Network Weights *positional* (312 pairs). Considering the top 1000 SNP pairs in each protocol, the most recurrent SNP pairs were rs5743293-rs2066844, rs7431710-rs4858795, and rs932826-rs4809335. These three pairs were detected in 13 protocols. All Epiblaster, linear regression, and BOOST runs identified these three SNP pairs.

Second, we studied the similarity network obtained from comparing the ranks of the top 1000 interactions using the Canberra distance (see Supplementary Section 3, Fig. 2b). This highlighted two extreme similarities: one between *functional* Epiblaster and linear regression and another between *positional* Epiblaster and linear regression (all for phenotypes corrected for multiple testing). Unlike the previous analysis, this rank-based comparison differentiated the inputs (i.e. *functional* or *positional*). To a lower extent, we observed similarities between Neural Network Weights, Epiblaster, linear regression, BOOST, MB-MDR, antEpiSeeker, and lightGBM (except lightGBM *positional* with phenotypes corrected for population structure). CASMAP (except CASMAP dominant *functional*) and epi-GTBN results were dissimilar to most other outcomes. Note that CASMAP produced results at the SNP-set level. That is, the output contained the region defined as $\{snp_{id1},... snp_{idN}\}$ and the $P$-value of the region. In our analysis, all pairwise interactions between SNPs in significant sets were considered, and the $P$-value of each interaction was set to the $P$-value of the region. This differs from how the interaction strength was computed in the other analyses. It produced many SNP pairs with the same $P$-values and, therefore, the same ranks.

### Beyond the variance: understanding and minimizing heterogeneity

The 34 epistasis results obtained at the SNP level were converted into gene-level results. Different methods require different ways to convert the results to the genes; we note that two GWAIS outputs were directly computed at the gene level via the Bayesian approach, and that two SNP-to-gene mapping strategies were applied for the linear regression outcomes (Section 2). Hence, in total, we generated 38 gene-level outputs.

After *functional* mapping (Section 2.1), there were no remaining pairs in the MB-MDR run without lower-order effect adjustment, the Bayesian model, and the epi-GTBN detection with ReliefF pre-functional SNP (corrected and uncorrected phenotypes). As a consequence, 34 gene-level SENs were considered for downstream processing. These networks included 5975 different gene pairs in total. Two interactions appeared in eight runs. *SLC22A4*-*SLC22A5* was identified only with *functional* protocols, using the following tools: AntEpiSeeker, CASMAP (recessive and dominant), Epiblaster (corrected and uncorrected), MB-MDR, BOOST, and linear regression. *AC108105.1*-*SNORA63* was detected via the same tools but only with the *positional* strategy. A total of 563 gene pairs were detected in at least two approaches. Gene pairs detected from *functional* protocols were often not included in the ones identified using the same tool but *positional* protocols. This result was expected since different SNPs were investigated: the *functional* input dataset was reduced *a priori* depending on the SNP to gene mapping and Biofilter gene pairs. Even when the same SNP pair is detected, the various strategies to obtain gene-level SENs from SNP-level SENs are likely to target different gene pairs. The different configurations considered in this work produced highly different results, highlighting the heterogeneity between protocols.

**Figure 3:**
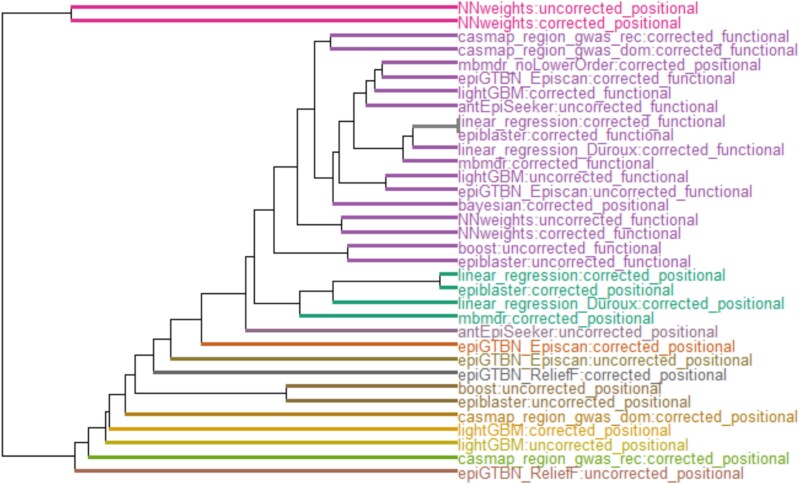
Clustering of gene-based SENs based on netANOVA [10] (Section 2.3.1). The input of the clustering algorithm is the list of gene epistasis networks obtained with the different protocols. Colors in the output dendrogram show the clusters identified by netANOVA.

We then used netANOVA [10] to analytically capture heterogeneity between clusters of SENs. A total of 13 clusters were detected (Fig. 3). The heatmap displaying the pairwise similarity between protocols is presented in supplementary Fig S1. The largest cluster contained 17 SENs, and the other clusters contained one to four networks (Fig. 3). When a cluster of size <3 is shown, it means that two smaller clusters in the same branch of the dendrogram (i.e. closer clusters) were identified as significantly different [18]. All the protocols involving a biologically driven strategy (*functional*) for SNP-to-gene mapping clustered together in the largest cluster. On top of these outcomes obtained from *functional* approaches, the *positional* Bayesian and MB-MDR without lower order adjustment runs were included in this cluster. The largest cluster was characteristic of small networks since SENs in that cluster on average only had 16 interactions (min 2, max 60), whereas the other SENs on average involved 410 pairs (min 26, max 1457). SENs also shared common gene pairs since a total of 167 gene pairs were identified in this cluster, and among them, 62 were detected in at least two approaches. Thus, the *functional* mapping strategy tended to homogenize SENs results. On the contrary, the *positional* protocols were split into many different clusters. For instance, epi-GTBN, CASMAP, and lightGBM were all in an independent cluster. The Neural Network Weights tool was furthest from other methods. Then, the cluster of four networks contained SENs obtained from linear regression, Epiblaster, and MB-MDR. This clustering was in line with results obtained from the ranked comparison of the top 1000 SNP pairs (Section 3.1). In summary, the heterogeneity observed with the gene-level analysis was mainly driven by the strategy for SNP-to-gene mapping rather than the tool used to detect epistasis. Importantly, heterogeneity was reduced when biological information was used.

Lastly, we formed a representative network. In particular, we aggregated the largest cluster of 17 SENs as described in Section 2. A total of 74 pairs from 100 nodes, forming 40 disconnected subnetworks, are obtained. The aggregated network and all gene-level SENs are visualized in Figure S2. In the aggregated network, the largest connected component contained 13 nodes, including many HLA-related genes (*HLA-A*, *HLA-B*, *HLA-C*, *HLA-E*, *HLA-F*, *HLA-G*). In the largest connected component, *LNPEP* and *ERAP1* were the nodes with the highest degree (7).

### Assigning biological relevance to the epistasis findings

#### SNP-level analyses

The first of most recurrent SNP-pairs derived from a simplistic co-occurrence analysis was rs5743293–rs2066844. SNP rs5743293 is located within the TLR5 gene, which plays a role in the innate immune response, particularly in recognizing bacterial flagellin. SNP rs2066844 is found in the NOD2 gene, critical for recognizing bacterial components and regulating immune responses, particularly in CD. TLR5 and NOD2 are involved in a complex network of physically interacting genes (Supplementary Fig. S3.) For the second highlighted SNP-pair, rs7431710–rs4858795, rs7431710 is located in the LEPR gene (leptin receptor), which is involved in energy homeostasis and body weight regulation, and rs4858795 is associated with the NPSR1 gene (neuropeptide S receptor 1), implicated in asthma susceptibility and immune regulation. Both genes LEPR and NPSR1 are involved in quite different physiological systems—energy regulation (LEPR) and immune response (NPSR1); we could not find widely established interactions between these two SNPs in the literature. Both SNPs were predicted to be regulatory ([43]). The third SNP pair identified by our SNP-level workflow was rs932826–rs4809335; rs932826 is located near the VEGFA gene (vascular endothelial growth factor A), relevant for blood vessel formation and involved in angiogenesis, and rs4809335 is linked to the CDKN1A gene (cyclin-dependent kinase inhibitor 1A), involved in cell cycle regulation and a key player in controlling cell growth and apoptosis. To our knowledge, no direct interaction evidence exists for this SNP pair. However, interactions between angiogenesis (VEGFA) and cell cycle regulation (CDKN1A) may be plausible given their roles in processes like cancer, where dysregulation of both blood supply and cell proliferation is common. Moreover, forming new blood vessels (VEGFA) is crucial during inflammation and wound healing. In IBD, chronic inflammation leads to tissue damage, and VEGFA is often upregulated as part of the body’s response to restore damaged intestinal tissues by increasing blood vessel formation [44]. CDKN1A (also known as p21) is important in regulating the balance between cell proliferation and apoptosis in intestinal epithelial cells, a key aspect in maintaining the intestinal barrier. Disruption in this balance is linked to mucosal injury and poor regeneration, which is one of the hallmarks of IBD. CDKN1A belongs is a member of the FOXO pathway, which was recently shown to be downregulated in UC patients [45]. Furthermore, rs932826 was predicted to affect ZBTB46 by CADD annotation via [43]. ZBTB46 defines and regulates ILC3s that protect the intestine [46]. We refer to Supplementary Section 8 for additional graphical annotation details.

#### Gene-level analyses

Integrating biological knowledge and aggregating homogeneous SENs are beneficial for adding biological relevance to GWAIS findings. The most recurrent gene pairs across all analyses (visualized in Fig. S2) were *SLC22A4*-*SLC22A5* and *AC108105.1* (novel transcript; ENSG00000287597) -*SNORA63* (RNA gene). The genes *SLC22A4* and *SLC22A5*, detected in eight *functional* analyses, were already identified as CD susceptibility genes. SNPs in the two candidate genes, both of which encode organic cation transporters, showed significant associations with CD [47]. SLC22A4 and SLC22A5 are involved in physical interactions via PDZK1 (see Supplementary Fig. S4), which was found downregulated in inflamed intestinal mucosa of UC patients [48]. To our knowledge, the genes *AC108105.1* and *SNORA63*, detected in eight *positional* analyses, were not previously identified as associated with IBD. In the aggregated network involving all the *functional* runs and two *positional* approaches, six HLA-related genes were underlined. Genome-wide significant epistatic signals within the MHC region, which include HLA-genes, were already detected [49]. Also, the hub *ERAP1* was previously reported as interacting with *HLA-C* in IBD in the Spanish population [50]. Hence, the aggregated SENs generated relevant and interpretable results, and the gene interactions obtained from different *functional* analyses were in line with the biology of IBD, whereas *positional* analyses identified multiple interactions that were hard to interpret.

### Simulation results

We applied six epistasis detection methods to evaluate their ability to identify causal SNP interactions in the simulated dataset. The tools included PLINK-LR (default logistic regression model), PLINK-BOOST, MB-MDR with and without codominant adjustment, EpiBlaster, and a LightGBM-based machine learning approach. For each method, we identified which of the predefined causal SNPs were successfully detected as interaction pairs. To assess the consistency between tools, we computed pairwise similarity scores based on the co-occurrence of top-ranked SNP pairs identified by each method. Finally, we applied netANOVA, to evaluate which tools exhibited the most similar detection patterns and to visualize relationships among epistasis detection strategies.

The assessment was based on the top 15 SNP pairs (Table 2) reported by each epistasis detection tool. PLINK-LR, PLINK-BOOST, and epiBlaster consistently detect two of the four true two-way epistatic pairs. In all three methods, the pair with the strongest statistical signal corresponds to the exponential interaction effect, while the pair with the second-strongest signal corresponds to the joint-dominant effect. MBMDR with codominant adjustment identifies the same two pairs, albeit in reverse order of importance. In contrast, MBMDR without codominant adjustment detects two pairs involved in the three-way interaction effect, both containing the SNP with a main effect. LightGBM successfully identifies all three-way interaction pairs as well as all two-way epistatic pairs, except for the one exhibiting a joint-recessive effect. These results indicate that the set of true interacting pairs is only partially recovered across methods, and that the tools provide complementary perspectives on the underlying epistatic architecture.

**Table 2:** Ranking of the epistatic SNP pairs detected by the different epistasis detection tools on simulated data. For each tool, the top 15 SNP pairs are included. The ranking of each interaction is reported in the corresponding cell, while empty cells indicate that the pair did not appear among the top 15. The *Effect* column reports the epistatic model effect as defined in EpiGEN

SNPa	SNPb	PLINK	PLINK Boost	MBMDR withAdj	MBMDR withoutAdj	EpiBlaster	LightGBM	Effect
rs10489136	rs6702469							joint-recessive
rs10492963	rs11801629	2	2	1		2	14	joint-dominant
rs16837624	rs6693450	1	1	2		1	10	exponential
rs17130482	rs1876839				3		15	triplet
rs17130482	rs6702469						7	triplet
rs1876839	rs6702469				2		12	triplet
rs4653522	rs6576939						11	multiplicative

The Jaccard analysis showed low to moderate overlap among tools (Figure S5). PLINK-LR and EpiBlaster had the highest similarity ($J = 0.50$), followed by PLINK-BOOST and MBMDR with codominant adjustment ($J = 0.30$). LightGBM and MBMDR without codominant adjustment showed minimal overlap ($J \leq 0.15$). NetANOVA clustering (Figure S6), which evaluates network-structure similarity among the epistasis detection tools, reflected the same groupings.

LightGBM recovered the highest proportion of true interactions ($40\%$), while the remaining tools recovered $~13\%$. Ensemble aggregation did not improve performance: the union contained $62$ edges ($six$ true interactions), the intersection contained none, and the LCA network reduced to $38$ edges with two true interactions, notably the pairs with the joint-dominant and exponential effects. We refer to Supplementary section 9 for further details.

### Factors shaping GWAIS protocol clustering

Clustered GWAIS protocols (section 3.1 and 3.2) can be further analyzed to reveal the key factors driving (dis-)similarities in epistasis results. We observed two main factors largely impacting cluster formation of GWAIS protocols: the type of modeling framework adopted by the protocol [51] and whether exhaustive or non-exhaustive screening for epistasis was performed [52].

#### Modeling framework

Cluster formation can be explained by having a similar underlying modeling framework. This was also observed via the co-occurrence network in Section 3.1, indicating that Epiblaster, BOOST, and linear regression produced the most similar results. The similarity between Epiblaster and linear regression was expected since Epiblaster (albeit involving an additional pre-filter step) is also based on linear regression. In addition, the main difference between BOOST and linear regression lies in how the interaction effect is tested. The linear regression tests interactions based on alleles ($\chi ^{2}$ test with $df=1$), whereas BOOST tests interactions based on genotypes ($\chi ^{2}$ test with $df=4$).

#### Input-level interventions

The dendrogram presented in Fig. 3 clearly shows the impact of exhaustive and non-exhaustive epistasis detection strategies [52].This was also seen by the resemblance network based on comparing ranked lists of outputs. Exhaustive protocols in our study were BOOST, linear regression, and MB-MDR. The non-exhaustive strategies were lightGBM, epi-GTBN, the Bayesian network approach, AntEpiSeeker, CASMAP, Epiblaster, and the Neural Network Weights approach. The non-exhaustive methods were two-stage approaches, where the first stage was a filtration step that reduced the search space. Filtration can be enhanced by artificial intelligence as in Neural Network Weights, lightGBM, Bayesian networks, antEpiSeeker.

Neural Network Weights, epi-GTBN, CASMAP, and lightGBM results were identified as the most dissimilar to other findings in the netANOVA procedure (Fig. 3). The ranked-based analysis (Fig. 2b) also highlighted that CASMAP and epi-GTBN results were very different from other outcomes. Notably, in epi-GTBN, feature selection was performed on the data with ReliefF and Episcan. As in the Epi-GTBN original paper, the SNPs presenting main effects were discarded, and the datasets were constructed considering the top 400 remaining variables only (Supplementary section 1), which generated a very limited SNP input space. CASMAP is perhaps the most different from the other tools, by construction, because it investigates entire SNP sets, but also because its filtration step is quite distinguishable from the other methods. CASMAP tests all possible sets of SNPs except the “non-testable” ones, where non-testable means that the minimum $P$-value that could be obtained with this set is larger than the significance threshold.

## Discussion

The workflow introduced in this study serves as a post-processing approach, facilitating the examination of how different GWAIS protocols influence final conclusions. By constructing and interpreting co-occurrence, resemblance, and gene-level SENs, the workflow provides insights into output consistency across protocols. SEN clustering and an aggregation algorithm further identify protocols yielding similar results, enabling meaningful data integration. Coupled with existing literature detailing analytic methodologies, our workflow aids in recognizing complementary protocols and reinforcing robust conclusions.

Existing reviews on epistasis highlight the variability in genome-wide association interaction study (GWAIS) protocols. Cordell *et al*. [53] focus on defining epistasis, while de Visser *et al*. [54] explore its potential causes. Aschard *et al*. [55] discuss conceptual differences and overlaps with genome-wide environment interaction studies, Niel *et al*. [52] review recent strategies for uncovering epistasis, and Van Steen and Moore [56] provide best practices for improving interpretability. Although simulations are widely used for benchmarking, their designs frequently encode method-specific assumptions, which restrict meaningful cross-tool comparisons. To address this, we analyzed both simulations incorporating realistic interaction structures and real-world datasets, aiming to capture heterogeneity as it naturally arises in practice. However, comparing these protocols remains challenging due to differences in focus, evaluation criteria, and the simulation data used. Additionally, many epistasis detection tools suffer from limited maintenance due to constrained academic resources, complicating their reproducibility.

In this study, we took a different approach, analyzing over 30 GWAIS protocols spanning various analytic paradigms and analysis interventions. These interventions occur at different levels: data input (e.g. filtering via biological knowledge), modeling/testing (e.g. confounder adjustment), and output processing (e.g. multiple testing correction). The protocols considered included likelihood ratio-based tests and parametric regression methods such as BOOST, AntEpiSeeker, CASMAP, linear regression, and Epiblaster. Bayesian methods were represented by epi-GTBN and the gene-based ”Bayesian network” approach. MB-MDR covered multidimensionality reduction techniques, while LightGBM and Neural Network Weights represented machine learning approaches. Our protocol selection was based on their analytical diversity, code availability, computational efficiency, and ease of implementation, drawing inspiration from [57–60].

To enhance the robustness of statistical epistasis analyses, interventions can be introduced at various stages of the analysis pipeline. These include input-level modifications (e.g. incorporating functional genomic annotations or restricting to tissue-specific genes), modeling adjustments (e.g. confounder correction or validation via permutation testing), and post-processing techniques such as integrating results across multiple detection tools. Consensus interactions identified through ensemble approaches can provide more reliable findings. However, post-processing interventions are often computationally intensive, requiring sophisticated techniques.

The conversion of SNP–SNP interactions into gene–gene interactions involves mapping SNPs to genes, aggregating interaction statistics, and applying multiple testing corrections. Our *functional* strategy restricted the analysis to gene pairs with plausible functional interactions, improving statistical power by reducing multiple testing burdens and enhancing biological interpretability. This approach produced results more consistent with known IBD biology than *positional* analyses, which yielded interactions that were often difficult to interpret. Biofilter was chosen for candidate gene pair selection due to its ability to highlight biologically meaningful interactions, although broader search spaces using HINT or STRING [61, 62] were beyond the scope of this study. The workflow is modular, and priors such as protein–protein interaction networks or tissue-specific co-expression modules could be incorporated at the SNP–gene mapping stage or during SEN aggregation to refine interpretability further.

Gene interactions identified via *functional* strategies tended to align better with known IBD biology, whereas *positional* analyses uncovered interactions with less clear interpretations. Notably, functional mapping reduced the SNP set available for epistasis detection by over half, affecting result detectability due to competing forces: a larger search space theoretically increases discovery, but multiple testing burdens counteract this effect. The biology-informed SNP set selection focused the analysis and often yielded nonoverlapping results compared with other protocols.

Concordance among epistasis detection methods in EpiGEN simulation was limited, with tools clustering according to their underlying methodology. Classical pairwise approaches grouped together, whereas LightGBM and MBMDR without codominant adjustment showed clearly distinct profiles, likely reflecting their ability to capture nonlinear or higher order interactions. LCA aggregation recovered two variant pairs, corresponding to joint-dominant and exponential EpiGEN effects. Incorporating additional tools with diverse decision boundaries may help LCA identify a larger fraction of the true underlying interactions.

Epistasis analyses remain computationally demanding, and our study deliberately worked within limited resources to draw attention to the overlooked challenge of heterogeneity in GWAIS results. While future advances such as federated approaches will help reduce computational barriers, our focus was on interpretability: by using SENs as both visualization tools and prioritization filters, the workflow emphasizes interactions consistently supported across protocols, thereby reducing method-specific artifacts while retaining sensitivity to diverse genetic architectures. This cross-method consensus provides a pragmatic balance that strengthens confidence in epistasis findings and enhances their translational potential.

Beyond SNP-set manipulation, phenotype handling also impacts epistasis detection. Epistasis tools vary in their treatment of binary versus continuous traits and confounder accommodation, directly influencing trait transformations and result interpretations. Furthermore, gene-level SENs may prioritize different genes compared with co-occurrence or resemblance networks due to their reliance on significance measures. While multiple testing corrections are essential for reducing false positives in GWAIS, they cannot fully address epistasis’s high-dimensional, nonlinear, and context-dependent nature. Computational strategies such as reducing the search space, employing efficient algorithms, and leveraging high-performance computing or quantum computing [63] can mitigate these challenges.

Different SENs need not be problematic if they provide complementary disease perspectives. As demonstrated in D’Silva *et al*. [64], multi-tool applications help establish consensus findings by converging on robust epistasis signals. In our work, we focused on SEN aggregation using binarized summary networks for similar GWAIS protocols. Weighted network aggregation preserves interaction strength, essential for biological insight, but involves complex computations. Alternative aggregation strategies, such as network diffusion-based approaches, rank product methods, or voting-based ranking (e.g. Borda count), offer potential solutions for enhancing result robustness, network density, and interpretability. However, optimal methods may differ across diseases, requiring further evaluation. Recommendations based on our IBD-specific workflow applications are detailed in Supplementary Section 11. We further confirmed the robustness of our results through a parameter sensitivity analysis, detailed in Supplementary Section 2, showing that moderate variations in thresholds for gene–gene pair retention, $P$-values, and minimum EpiGTBN runs did not substantially alter network clustering or the grouping of biologically driven strategies

Interpreting statistical network findings in a biological context remains challenging. Network-driven enrichment analysis can address this issue by analyzing gene wiring patterns. Dense subnetwork modules in SENs may represent functional biological modules or pathways, even if individual interactions show weak disease associations. This approach transcends pathway gene listing by leveraging gene interactions to uncover novel regulatory mechanisms. The chosen GWAIS protocol also affects expected SNP-level and gene-level network densities, as protocol-specific features (e.g. intermediate phenotypes or two-locus effect testing) influence output structure. Moreover, network-guided strategies could enhance drug discovery by identifying system-level genetic interactions rather than single-gene targets, increasing therapeutic potential.

## Conclusion

Different analytical tools for epistasis detection come with distinct assumptions, strengths, and limitations, making direct comparisons challenging and often leading to inconsistencies. While integrating evidence from multiple methods can enhance robustness and interpretability, not all combinations of methods or results are meaningful. Our workflow provides an alternative approach to evaluate and interpret the impact of various GWAIS analysis protocols on the results. Additionally, it offers guidance on which results to integrate and which to maintain as complementary perspectives. Although illustrated here using IBD as a representative complex trait, the workflow is phenotype-agnostic and can be readily extended to simulated datasets or other conditions, provided appropriate genotype–phenotype data and computational resources are available, enabling future assessments of sensitivity, specificity, and generalizability. Looking ahead, increased accessibility to computational resources, improved interoperability between tools, and standardized analysis pipelines could foster multi-tool approaches to strengthen robustness in future studies.

**Key points**

- Different epistasis detection tools have varying assumptions and limitations, leading to inconsistent results and making direct comparisons challenging in GWAISs.
- The study introduces a workflow for comparing SENs across different protocols, enhancing result reliability and enabling result aggregation for improved consistency.
- Integrating prior biological knowledge and applying interventions such as confounder adjustments or cross-validation improve robustness and interpretability of epistasis findings.
- Improved computational resources, better tool interoperability, and standardized pipelines are needed to promote multi-tool approaches, strengthening the reliability and interpretability of GWAIS findings in complex diseases.

## Supplementary Material

Suppl_Turning_heterogeneity_of_statistical_epistasis_networks_to_advantage_bbaf699

## Data Availability

Simulations are generated with EpiGEN (https://github.com/daisybio/epigen ), and are available on GitHub at https://github.com/DianeDuroux/SENs. The dataset underlying this article is available upon request from the International Inflammatory Bowel Disease Genetics Consortium (https://www.ibdgenetics.org/). The code supporting this study is openly available on GitHub.com at: https://github.com/DianeDuroux/SENs. To facilitate reproducibility and ease of use, we provide a Docker container that includes all required dependencies and configurations. This ensures that analyses can be replicated across different computing environments without additional setup. Instructions for installation and execution are provided in the repository’s README file.
