# Supplementary material for: Turning heterogeneity of statistical epistasis networks to an advantage
Source: Brief Bioinform. 2026 Jan 19;27(1):bbaf699. doi: 10.1093/bib/bbaf699 (PMC12814973; doi:10.1093/bib/bbaf699)

Turning heterogeneity of statistical epistasis networks to advantage

Diane Duroux [1,+], Federico Melograna [1,2,+,*], Héctor Climente-González [3], Bowen Fan [4,5], Andrew Walakira [6], Edoardo Efrem Gervasoni[7], Zuqi Li [2], Damian Roqueiro [4,5], Fabio Stella [7], and Kristel Van Steen [1,2]

[1]BIO3 - GIGA-R Molecular and Computation Biology, University of Liege, Place du 20 Août 7, 4000 Liège, Belgium; [2]BIO3 - Department of Human Genetics, KU Leuven, Herestraat 49,B-3000 Leuven, Belgium; [3]RIKEN Center for Advanced Intelligence Project (AIP), [4]Department of Biosystems Science and Engineering, ETH Zurich, Basel 4058, Switzerland; [5]SIB, Swiss Institute of Bioinformatics, Switzerland; [6]Centre for Functional Genomics and Bio-Chips, Institute for Biochemistry and Molecular Genetics, Faculty of Medicine, University of Ljubljana, Ljubljana, Slovenia; [7]Department of Informatics, Systems, and Communications University of Milano-Bicocca, Italy; [+]Authors share first authorship;

Supplementary Material

1 Description of applied software and methods for epistasis detection

The applied GWAIS methods cover a broad spectrum of modelling and testing paradigms. The difference in paradigms can also translate into differences in the input required. Only part of the tools accepts covariates [1, 2] or handle continuous phenotypes [1, 3, 4, 5, 6, 7]. The input of Epi-GTBN was pre-filtered using ReliefF and Episcan for computational reasons. In the Bayesian model, the algorithm was applied to the top 2,000 genes identified by ReliefF. Finally, some methods could not handle missing values and required imputed data [2, 5, 6, 7, 8, 9]. In this project, SNP data were imputed using the function knn.impute of the bnstruct package in R [10], under the assumption that data are missing at random (i.e., follow a so-called MAR missingness process).

Depending on the analytical model, we obtained diverse types of outputs (see Table S1). One difference is the genetic unit of analysis. We obtained outcomes at the level of SNP-pairs [1, 3, 4, 5, 6, 8, 9, 11], SNP-sets [2], and gene-pairs [7]. Since CASMAP produced lists of SNPs and their associated P-value, an additional post-processing step was applied. We did not consider regions with only one SNP (main effect), and all the possible interactions were considered for the detected

*To whom correspondence should be addressed. Email: federico.melograna@kuleuven.be

Table S1: Highlighted output properties of the different epistasis detection algorithms as applied to real-world data. Considered are the units of analysis that drive the level at which epistasis results are presented, the strategy adopted to assess the strength of the epistasis findings, and whether or not the presented findings are adjusted for multiple tests.

Method	Level	Output Type	Multiple testing correction
AntEpiSeeker [9]	SNP	P-value	Yes
Bayesian model [7]	Gene	Posterior inclusion probability	No
(PLINK's) BOOST [11]	SNP	P-value	No
CASMAP [2]	SNP-set	P-value	Yes
Epiblaster [5]	SNP	P-value	No
Epi-GTBN [8]	SNP	# occurrence	No
LightGBM [4]	SNP	Interaction strength	No
Linear Regression [3]	SNP	P-value	No
MB-MDR [1]	SNP	P-value	Yes
Neural Network Weights [6]	SNP	Rank	No

region of at least two SNPs. The P-value of the entire set was given to all the internal pairwise interactions. The results of the Bayesian model, directly obtained at the gene level, were only filtered based on the Biofilter networks (see Section **??**) for the *functional* dataset. Furthermore, the value associated with the epistatic loci can be of different nature. Interactions can be ranked through a value indicating how strong the link is [4, 6, 7, 8]. They can also be linked to a statistic or a P-value [1, 2, 3, 5, 9, 11]. Even if there is a significance testing strategy, only a portion of the tools include multiple testing corrections when deriving the P-values [1, 2, 9].

Here, we first describe the ideology behind the adopted methods. Extra details and a summary of key distinctions between approaches are given in Table. Second, we provide additional information about the methods as practically used in this work (including mentioning of parameters and options selected).

PLINK Linear regression:

- Description: PLINK regression is one of the oldest tool to detect epistasis. This test is applicable for population-based samples. It uses either linear or logistic regression, depending on whether the phenotype is a quantitative or binary trait. PLINK makes a model based on allele dosage for each SNP. This test therefore only considers allelic by allelic epistasis. All pairwise combinations of SNPs can be tested. The output consists on pairwise epistatic results above a certain significance value along with a summary of all the pairwise epistatic tests (e.g. maximum test, proportion of tests significant at a certain threshold, etc). The software offers multiple options such as "epi1" which adjust the P-value for inclusion of pairs in the main report, "epi2" which adjust the P-value threshold for qualification as "significant epistatic test results".

- Application: We used PLINK 1.9 to detect epistasis through a linear regression on the population structure adjusted phenotypes with the option `--epistasis`: $Y = \beta_0 + \beta_1 g_A + \beta_2 g_B + \beta_3 g_A g_B$, where g_A and g_B are the genotypes under additive encoding for SNPs A and B respectively, Y is the adjusted phenotype, and β_0, β_1, β_2, and β_3 are the regression coefficients. PLINK performs a statistical test to evaluate whether $\beta_3 \neq 0$. It only returns SNP pairs with a P-value lower than a specified threshold. We used the default 0.0001.

MB-MDR:

- Description: MBMDR is a software that is able to identify genetic interactions for a variety of SNP-SNP based epistasis models. The algorithm exhaustively explores the association between each SNP pair and the phenotype, using all available cases. The method is non-parametric in the sense that no assumptions are made regarding the modes of interaction inheritance. MB-MDR is fairly robust to deviations from the normal distributions for Y, even though the final MB-MDR test for non-binary traits is by default the result of a sequence of t-tests. Type I error is controlled with the rank-based multiple testing corrections (gammaMAXT [1]). The Model-Based part of MB-MDR assumes the default of adjusting two-locus testing for main effects (SNP A, SNP B) and thus the considered MB-MDR alternative hypothesis was H1: the joint effect of SNP A and SNP B goes beyond additive single SNP effects.
- Application: We used MBMDR 4.4.1 to detect epistasis. We explored association between SNP pairs and the phenotype adjusted for population structure. We used the parallelized version with default options, including gammaMAXT correction for multiple testing. A first run was applied with codominant adjustment and a second run was performed without adjustment for lower order effect. Note that no lower order effect correction is likely to reveal many SNP-pairs, some of which due to strong main effects rather than epistatic effects.

PLINK BOOST:

- Description: Boolean Operation-based Screening and Testing" (BOOST) uses the exhaustive search to find epistatic interactions in GWAS. The authors designed a Boolean representation of genotype data, which promotes not only space efficiency but also CPU efficiency because it involves only Boolean values and allows for the use of fast logic (bitwise) operations to obtain contingency tables. On the basis of this data representation, they propose a two-stage (screening and testing) search method. In the screening stage, a non-iterative method is used to approximate the likelihood ratio statistic in evaluating all pairs of SNPs and select those passing a specified threshold. Most non-significant interactions will be filtered out, and the survival of significant interactions is guaranteed. In the testing stage, we employ the classical likelihood ratio test to measure the interaction effects of selected SNP pairs. Hence, BOOST tests interactions based on genotypes, using the Chi-2 test with df=4.)
- Application: We applied the PLINK implementation of BOOST –fast-epistasis boost with default options on the case control phenotypes.

lightGBM:

- Description: LightGBM is short for light gradient boosting machine. It is based on decision tree algorithms and frequently developed for classification, regression and other machine learning task. The algorithm focus is on both performance and scalability. The decision tree is a powerful tool to discover interaction among independent variables. Variables that appear together in a traversal path are interacting with one another, since the condition of a child node is predicated on the condition of the parent node. To detect interactions, one could take a recusive walk through each individual decision tree and extract the information gain between the parent-child node as the interaction between these two features in the specific tree. Then the final interaction terms are calculated by aggregating over all the trees.
- Application: We applied lightGBM algorithm on both phenotypes adjusted for population structure and case controls. In the output, the mean variable is indicative of the ranking. Mean is the aggregation of the interaction detected from 3 different lightGBM models: the smaller the mean, the stronger the interaction.

Epi-GTBN:

- Description: The epi-GTBN algorithm firstly construct the network of gene loci for specific phenotype using Bayesian network, and then mine the epistasis for specific phenotype. However, due to Bayesian network usually uses the partial or random search strategy, it is easy to fall into local optimum and further to influence the learning accuracy. Genetic algorithm has the characteristic of rapid global search and avoiding falling into local optimization. In this approach, the authors use the genetic algorithm into the heuristic search strategy of Bayesian network. The evolution of individual structure is realized through three genetic operations (selection, crossover, mutation), and thus to find the optimal network structure. Inspired by the genetic tabu algorithm used in [12], they use the tabu search strategy into the crossover and mutation operation of genetic algorithm. It can help to enhance the diversity of population and thus to obtain the global optimal solution. In the genetic algorithm, the quality of the initial population has an important effect on the result. We use mutual information entropy calculation method to calculate the relationship between gene loci and phenotype, and thus to construct the initial network. It can help to enhance the quality of the initial network. In order to speed up the calculation, we convert the genotypic data into binary Boolean data and then directly carry out the fast logic (bitwise) operation to calculate the mutual information.
- Application: We used the Epi-GTBN R library version 2.1.0-3. The main function of Epi-GTBN library that was used is gtbn2, which allows to mine for 2-loci epistasis. Epi-GTBN was applied on both case-control phenotypes and phenotypes adjusted for population structure. Firstly, feature selection was performed on the data with ReliefF and Episcan. For Episcan, we constructed the list according to the first appearance of each SNP in the pairs. Then, as in the Epi-GTBN paper, the SNPs presenting main effects (i.e. those for which the test

for conditional independence between them and the phenotype variable had P-value lower than 0.01) were removed from the lists, and the datasets were constructed considering the top 400 variables remaining. Epi-GTBN was applied on unimputed data. Since missingness is not handled, missing observations were removed. Moreover, in order to achieve reliable results from Epi-GTBN, a Bootstrap approach was applied as suggested in multiple studies [13, 14, 15]. It allows to evaluate connections confidence in Bayesian networks by generating perturbed datasets, applying the structure learning algorithm on them and then considering the networks that have been built. These networks will present changes in their structures since they were generated from slightly different data. In this approach, such perturbations are carried out by random sampling the observations with replacement. We created 10 perturbed datasets, and Epi-GTBN was applied on each of them by randomly sampling 10,000 observations. The outputs contain the number of times each relation between two SNPs appears among the 10 networks. The more a pair appear in these 10 networks, the stronger the association.

Epiblaster:

- Description: Epiblaster is a two-stage parametric algorithm that uses the combination of quasi-likelihood and linear model (both lm and logistic regression, depending on the output) to have a ranked list of SNPs with the attached score and adjusted P-value. The first step is computed via a filtering stage using the difference of Pearson's correlation coefficient to have a fast and accurate ranking of all the SNP pairs; it is an exhaustive method. Then, for the second step, only top-k pairs are selected. On these pairs, the real P-value and test statistic are computed via a more accurate lm model with a real likelihood. The collected statistics for the rank are the P-value resulting on a lm model with the phenotype as outcome and as input the marginal SNPs that we want to take into account and the interactions. The P-value associated with this Beta is the measure of the association, and since multiple testing is performed the adjustment is needed.

- Application: Epiblaster was applied on both case control phenotypes and phenotypes adjusted for multiple testing. The second step was not available and had been custom implemented for the work. In this step, the top 10,000 pairs were selected.

Neural Network Weights:

- Description: MLM-M is a method that combines neural network and explainability in order to find a outliern-ish value for the SNPs-pairs. The building block is a fully-connected NN with a fixed number of neurons. The input are all the SNPs and the output is the outcome. The activation function is ReLU for every layer except the final one. Depending on the outcome a categorical cross entropy or a MSE is used. The optimizer is ADAM. The computation of the epistasis SNPs is done via considering the SNPs that have the highest weights, so the highest interactions, jointly in one Neuron of the first layer. Iterating this for all the neurons in the first layer, and averaging the results via a neuron-importance weight, it is possible to have an ordered list of SNPs pairs that jointly have a great effect on the outcome.

- Application: The algorithm was applied on both case control phenotypes and phenotypes adjusted for multiple testing. 4 layers with 140,100,60,20 neurons each was applied, a main effect net active, and the following splitting: 80% training, 10% validation 10% test. The result is top 10,000 ranked list with SNP-pairs and an associated outliernish value.

Bayesian model:

- Description: This pipeline takes GWAS SNP data as input, develops gene-level summaries via diffusion kernels on graphs, and uses a Bayesian framework and kernel principal components as new units of analysis to discover genes and gene-gene interactions in relation to a complex trait. SNPs are first mapped to their genes, and "gene files" extracted for each gene separately. Then kernel principal components (KPC) are calculated for each gene. The first principal component is used as a gene summary and thus become the representations of the genes. The kernels are then used in the model described by Antonelli et al., 2020 to yield posterior inclusion probabilities (PIP) for the gene-gene interactions. The higher the PIP, the stronger the assocation.

- Application: The model was applied on phenotypes adjusted for population structure. The algorithm is applied on the top 2,000 gene identified by ReliefF. An interaction with a PIP greater than zero is considered significant.

AntepiSeeker:

- Description: AntEpiSeeker is based on the ant colony optimization (ACO) paradigm. As the name suggests, it was inspired by how ants find the shortest path between a source of food and the colony. Ants lay chemical signals (pheromones) as they move, and the shortest paths will be traversed more often, and accumulate more pheromones over time. In ACO, artificial ants are parallel agents that communicate through a probability mass function (PMF), containing the probability of picking a path. When an ant picks a specific path, it increases its probability. On the other hand, all paths lose probability at a constant rate, so the probability of the ones that are not selected will tend to 0. In AntEpiSeeker, the ants select sets of SNPs of the user-specified size. The probability of picking up a SNP is governed by a PMF, updated as described above. At the beginning, all SNPs have the same probability of being selected. Then, the association between the SNP set picked by each ant and the phenotype is measured via a χ^2 test. Based on that, AntEpiSeeker updates the probability of each SNP: the larger the χ^2 of the sets that include a SNP, the larger the update. On a second stage, AntEpiSeeker exhaustively looks for epistasis between the top scoring SNPs.

- Application: Available implementation of AntEpiSeeker was applied on the data with phenotypes adjusted for population structure. The output file records all detected epistatic interactions with significant user-defined p value threshold (after the Bonferroni correction). AntEpiSeeker report all detected epistatic interactions at a P-value threshold. We compared

the top-1,000 pairs in Fig 2. In addition, AntEpiSeeker incorporates a procedure for minimizing false positives that we used to further reduce the SNP-pair set for the construction of the gene-level SENs. The procedure build a set of epistatic interactions with minimized false positives by removing SNP pairs containing a SNP present in other pair with a lower P-value.

CASMAP:

- Description: CASMAP tests the association between sets of SNPs (i.e. regions) and the phenotype. Testing genomic regions for association is based on the hypothesis that aggregating multiple neighboring SNPs will yield a stronger signal. Regions are not specified a priori, instead, it tests all possible sets except the "non-testable" ones. Hence, it exploit the concept of testability (Tarone, 1990) and implement an efficient pruning criterion in a branch-and-bound fashion (Terada et al., 2013). Non-testable means that the minimum pvalue that we could obtain with this set is larger that the significance threshold. An extreme example is when all the SNPs considered have value 0. It is computationally intensive but it is useful to decrease the multiple testing burden.

- Application: CASMAP was applied on the binary phenotypes and included the first 7 PCs to correct for population structure. The region-GWAS analysis on CASMAP was ran on two different encodings of the data: dominant and recessive. Each output file contains the region defined as {snp_id1, ... ,snp_idN} and the P-value of the region. In our analysis, all pairwise interactions between SNPs in significant sets were considered.

A summary of the key differences between the tools is given in Table S2 and addresses the following features: underlying methodology, SNP encoding, phenotype flexibility, confounder adjustment, computational efficiency, ability to detect higher-order interactions.

2 Parameter Sensitivity Analysis

We extended our framework with a parameter sweep on the cutoffs used to retain gene–gene pairs. The impact of applying different thresholds on the number of gene pairs retained by each epistasis detection approach is shown in the figure below. The "Baseline" column corresponds to the parameter settings used in the manuscript, while the other columns reflect results obtained with the alternative parameter values.

Relative cutoffs. For NN, we compared the baseline threshold of $0.7 \times \max(\text{weight})$ with $0.5 \times \max(\text{weight})$ and $0.9 \times \max(\text{weight})$. For LightGBM, we analogously evaluated $0.7 \times \max(\text{strength})$, $0.5 \times \max(\text{strength})$, and $0.9 \times \max(\text{strength})$. In the unfiltered setting, relaxing the cutoff to $0.5\times$ substantially increased the number of retained pairs, from a few hundred to $> 11{,}000$ for NN and from ~ 600 to $\sim 1{,}000$ for LightGBM. The filtered setting was less sensitive to this relaxation. Conversely, tightening the cutoff to $0.9\times$ markedly reduced yields; in the filtered setting,

Table S2: Key characteristics of the epistasis algorithms and their ability to handle various input data are highlighted. The fifth column specifies the method used by each algorithm to reduce computational complexity, while the sixth column indicates whether the algorithm is capable of detecting higher-order interactions (i.e., beyond pairwise).

Method	SNP Encoding	Phenotypes	Confounder Adjustment	Computational Burden Reduction	Higher-order Interactions
Plink Linear Regression [3]	Additive/ dominant/ recessive	Binary, continuous	Covariates in regression model	Efficient matrix operations	Only pairwise
MBMDR [1]	Discrete categories	Binary, continuous (extensions)	Permutation-based or mixed models	Group comparisons, permutation testing	Yes (2-way, 3-way, etc.)
Plink Boost [11]	Binary or ordinal	Binary	Covariates in boosting model	Gradient boosting	Pairwise, but higher possible
LightGBM [4]	Numerical/ categorical	Binary, continuous	Covariates in decision tree	Gradient-based sampling	Yes (through tree splits)
Epi-GTBN [8]	Categorical	Binary, continuous	Bayesian conditioning	Priors to reduce search space	Yes
Epiblaster [5]	Additive or dominant	Binary	Covariates in GLM	Efficient pairwise interaction testing	Limited to pairwise
Neural Network Weights [6]	Continuous (0,1,2)	Binary, continuous	Covariates as input features	SGD, mini-batch processing	Yes (complex, higher-order)
Bayesian Model [7]	Categorical/ continuous	Binary, continuous	Bayesian framework	Priors, model selection	Yes (high comp. cost)
AntepiSeeker [9]	Decision tree-based	Binary, continuous	Covariates in decision tree	Tree-based algorithms	Yes (tree-based splits)
CASMAP [2]	Combinatorial	Binary, continuous	Covariates in combinatorial framework	Combinatorial optimization	Yes (combinatorial methods)

Table S3: Details of the implementation of the epistasis algorithms, highlighted the software and the main function to perform the epistasis analysis. The sixth column indicates whether a parallelized version is available. The seventh column indicates whether results on synthetic data are available.

Acronym	Authors	Software (version)	Implementation (URL)	Main function(s)	Parallel execution provided	Results synthetic data
Linear Regression	Purcell et al.[3]	PLINK 1.9	cog-genomics.org/plink/	epistasis	Yes	No
MB-MDR	Lishout et al. [1]	MBMDR 4.4.1	bio3.giga.ulg.ac.be/ /	./mbmdr	Yes	Yes
PLINK Boost	Wan et al.[11]	PLINK 1.9	cog-genomics.org/plink/	boost	Yes	Yes
lightGBM	Ke et al. [4]	lightGBM 3.3.2	lightgbm.readthedocs.io	lightgbm	Yes	No
Epi-GTBN	Guo et al.[8]	epi_GTBN 2.1.0-3	github.com/Epi-GTBN/package	gtbn2	No	Yes
Epiblaster	Kam-Thong et al. [5]	episcan 0.0.1	rdrr.io/cran/episcan/	epiblaster1geno and epiHSIC1geno	Yes	Yes
Neural Network Weights	Tsang et al. [6]	NID	mtsang/neural-interaction-detection	train/get_weights/ get_interactions	No	Yes
Bayesian model	Walakira et al. [7]	kPCAepistasis	github.com/awalakira/kPCAepistasis	no main function	Yes	No
AntEpiSeeker	Wang et al. [9]	AntEpiSeeker1.0	nce.ads.uga.edu/ romdhane/AntEpiSeeker	./AntEpiSeeker	Yes	Yes
CASMAP	Llinares-López et al. [2]	CASMAP 0.6.1	github.com/BorgwardtLab/CASMAP	region_gwas *execute*()	No	No

Table S4: Number of significant hits with baseline parameters and using less and more stringent thresholds.

Epistasis detection approach	Baseline	P-value cutoff= 0.01	Relative cutoff=0.5	Relative cutoff=0.9	Min epiGTBN=1	Min epiGTBN=3
NNweights corrected unfiltered	185		11378	7		
NNweights noncorreted unfiltered	379		17817	6		
NNweights top10000 corrected filtered	17		48	3		
NNweights top10000 notcorrected filtered	31		49	4		
lightGlBMfiteredCorrected	3		5	1		
lightGBMfiteredUncorrected	5		6	5		
lightGBMunfiteredCorrected	590		1013	199		
lightGBMunfiteredUncorrected	610		1000	242		
antEpiSeeker$_{filtered}$	5	5				
antEpiSeeker unfiltered	86	86				
casmap, region gwas, dom, unfiltered all interactions	1069	838				
casmap, region, gwas, dominant filtered all interactions	28	26				
casmap region gwas rec. unfiltered all interactions	1457	1457				
casmap region gwas, recessive filtered all interactions	18	15				
mbmdr gammaMaxt filtered	7	7				
mbmdr gammaMaxt lowerOrder unfiltered	2	2				
mbmdr gammaMaxt unfiltered	66	66				
epiGTBN Episcan corrected filtered	2				6	0
epiGTBN Episcan, corrected unfiltered	239				6218	20
epiGTBN Episcan notcorrected filtered	8				31	1
epiGTBN Episcan notcorrected unfiltered	203				4774	22
epiGTBN Relieff corrected unfiltered	325				7794	17
epiGTBN ReliefF notcorrected unfiltered	344				7465	34

all approaches retained fewer than 10 pairs, with the exception of LightGBM in the unfiltered scenario.

P-value cutoffs. For MBMDR, CASMAP, and antEpiSeeker, the baseline analysis applied a p-value threshold of 0.05. We compared this with a more stringent cutoff of 0.01. This adjustment had no effect on the number of retained pairs for MBMDR or antEpiSeeker, while CASMAP showed a slight reduction in the number of detected pairs.

EpiGTBN runs. For epiGTBN, the baseline criterion considers a pair significant if it appears in at least 2 runs. We tested alternative thresholds of 1 (less stringent) and 3 (more stringent). Lowering the threshold to 1 substantially increased the number of retained pairs (to several thousand), while raising it to 3 reduced the number considerably (to only a few dozen). Under the baseline of 2 runs, the filtered scenario yielded a few hundred pairs, striking a balance between sensitivity and stringency.

The number of significant hits for the various cut-offs are reported in Supplementary Table S4.

2.1 Impact on cluster stability and biological interpretation

We then examined how different threshold choices affect cluster stability and biological interpretation. Using netANOVA to cluster similar SENs, we compared three settings, shown in Supplementary Table S5. (i) Baseline (intermediate thresholds), (ii) the most stringent thresholds (p-value 0.01, relative cutoff 0.9, and minimum epiGTBN runs=3), and (iii) the least stringent thresholds (p-value 0.05, relative cutoff 0.5, and minimum epiGTBN runs=1).

The number of clusters was comparable for the relaxed (12) and intermediate (13) settings, both

Table S5: Effect of threshold variation on SEN clustering. Relaxed: p-value 0.05, relative cutoff 0.5, minimum epiGTBN runs 1; Intermediate (baseline): p-value 0.05, relative cutoff 0.7, minimum epiGTBN runs 2; Stringent: p-value 0.01, relative cutoff 0.9, minimum epiGTBN runs 3.

Threshold	# Epistasis clusters	# SENs in largest cluster	All biologically-driven strategies in same cluster
Relaxed	12	17	yes
Intermediate (baseline)	13	17	yes
Stringent	9	19	yes

yielding a largest cluster of the same size (17). With stringent thresholds, the number of clusters decreased slightly (9), while the largest cluster grew (19). Importantly, across all threshold choices, the biologically driven strategies consistently clustered together, indicating that their grouping is robust to threshold variation.

To strike a reasonable balance between retaining a sufficient number of gene pairs while avoiding excessive inclusion, we recommend using thresholds such as a relative cutoff of 0.6–0.8, a replicability limit (e.g., minimum EpiGTBN runs) of at most 2, and a p-value threshold around 0.05. Our choice of thresholds was guided by two considerations: (i) consistency with prior network-based epistasis studies, where relative cutoffs have been widely used to retain the strongest signals while controlling network density; and (ii) preliminary exploratory checks, which indicated that moderate variation of the cutoff did not qualitatively alter the clustering structure or main conclusions.

3 Comparing epistasis outputs using SNP-level rankings and resemblance networks

All GWAIS tools or protocols generate measures that quantify the relevance of genetic interactions, such as P-values, test statistics, or importance scores. These measures, however, do not always directly indicate statistical significance, and deriving statistical significance often involves substantial computational effort. Nevertheless, a ranking of interactions based on these measures can always be produced. We initially compared outputs without incorporating network-based information to evaluate epistasis results at the SNP level.

Specifically, we first focused on the top-k ranked SNP interactions, as the most significant SNP pairs appear at the top of these lists, and set $k = 1{,}000$. Supplementary section 1 gives explicit details about how the ranked lists were obtained for each tool or protocol. Second, the ranked lists were subsequently used to formally compare the output of protocols. Comparison was performed by counting the number of SNP pairs in common between protocols (i.e. co-occurence of SNP pairs between protocol outputs). Note that for protocols producing only $n < k$ interactions, we only compared these n interactions. We furthermore used the algorithm presented in Manduchi et al. [16], explicitly using ranks. It is based on computing the similarity of two protocols by comparing

ranked output lists with Canberra-based distance metrics. Adapted to our context, the distance between two ranked lists σ and τ of epistasis results was defined by

$$Ca^{k+1}(\sigma, \tau) = \sum_{SNP} \frac{|min[\tau(SNP), k+1] - min[\sigma(SNP), k+1]|}{min[\sigma(SNP), k+1] + min[\sigma(SNP), k+1]}$$

where "SNP" varies across all SNP pairs. As a consequence of this definition, the difference between two ranked lists represents the variations in the lower portion of the list less than those in the top. Note that when protocols identified fewer than k interactions (i.e., $n < k$), the rank of a pair not in the output was set to $(\#pairs \in output) + 1$. For instance, if one tool only outputted 67 SNP pairs, all the other pairs would have a rank of 68. Third, we used the function $Similarity = \frac{1}{1+Distance}$ to compute similarities from the aforementioned Canberra-based distances and created a resemblance network of protocols. The network's nodes represent protocols; edge widths represent the strength of similarity between protocols. Likewise a co-occurence network was constructed; the larger the number of top SNP pairs shared between two protocols, the thicker the corresponding edge in the co-occurrence network.

4 NetANOVA

It computes pairwise similarities between networks and uses them in unsupervised hierarchical clustering to obtain a dendrogram displaying potential groups. Processing from the top to the bottom of the dendrogram, netANOVA performs recursive tests for the distance between two groups of graphs to derive the cutoff that sets the number of clusters. Significance is assessed with the permutation of similarities between graphs. In this paper, we computed the distance between each pair of networks using DeltaCon distance [17] because the correspondence between the nodes (i.e. genes) across the networks was known. The number of permutations in the netANOVA algorithm was set to 9999, and the minimum number of networks per group was set to 3. The default values were considered for the remaining parameters: the method to compute the distance between each cluster in the hierarchical clustering (complete), the method to correct for multiple testing (depth of the dendrogram), the maximum P-value for 2 groups of networks to be considered as significantly different (0.05), the percentage of values permuted in the distance matrix (20%), and the seed for replicability (2021).

5 poLCA

The R function *poLCA* allows a dataset to be partitioned into exclusive groups called *latent classes*. The main latent class model is $P(y_n|\theta) = \sum_{j=1}^{S} \pi_j P_j(y_n|\theta_j)$ where y_n refers to one of N ($1 \leq n \leq N$) observations (i.e. edges) and the number of levels of y_n corresponds to the number of SENs to aggregate (i.e., similar networks belonging to the same cluster), with S the number of clusters (2: edge is present or absent), and π_j the prior probability (random) of belonging to cluster j. P_j

Table S6: Number of nodes, edges and clusters with different aggregation methods: LCA (default), intersection and union.

Method	Nodes	Edges	Clusters
Union	211	167	81
Intersection	0	0	0
LCA	100	74	40

is the cluster-specific probability of y_n given the cluster-specific parameters θ_j. The Expectation-Maximization algorithm was used to maximize the latent class mode log-likelihood function. The output was a vector of predicted cluster memberships for each edge. Since SENs are usually sparse, we set the larger group to *absent*.

6 Latent class analysis

We selected latent class analysis (LCA) as a principled method to consolidate similar SENs while preserving complementary information, under the assumption that networks can be partitioned into latent clusters characterized by shared interaction patterns. Simpler aggregation schemes, such as strict intersections or unions of SNP-pair results, are indeed easier to implement. However, these approaches risk either discarding valuable signals (intersections) or retaining excessive noise (unions).

To illustrate this, we have included a comparison of aggregated networks obtained using LCA, intersection, and union approaches, reporting the number of nodes, edges, and clusters in Supplementary Table S6 . As expected, the union produced the largest representative network (211 nodes, 164 edges and 81 disconnected subnetworks), whereas the intersection did not retain any nodes or edges. LCA yielded an intermediate solution, resulting in a network with 100 nodes, 74 edges, and 40 disconnected subnetworks. Hence, LCA provides a probabilistic framework that supports partial membership and uncertainty, thereby maintaining sensitivity to complementary signals while reducing false positives.

7 Gene-level SENs

We used the function mapIds to map Ensembl gene ids to symbol gene ids. This conversion tends to reduce the number of pairs gene pairs identified and was applied for readability. For the same reason, gene names are indicated when the SENs contains a maximum 20 genes. All the plot are visualized on the same base circle with fixed gene location to make them more easily comparable.

The heatmap Fig. S1 shows the similarities between gene-level SENs. The associated similarity matrix is the input of the netANOVA worflow.

Fig. S1: NetANOVA clustering of gene-based SENs

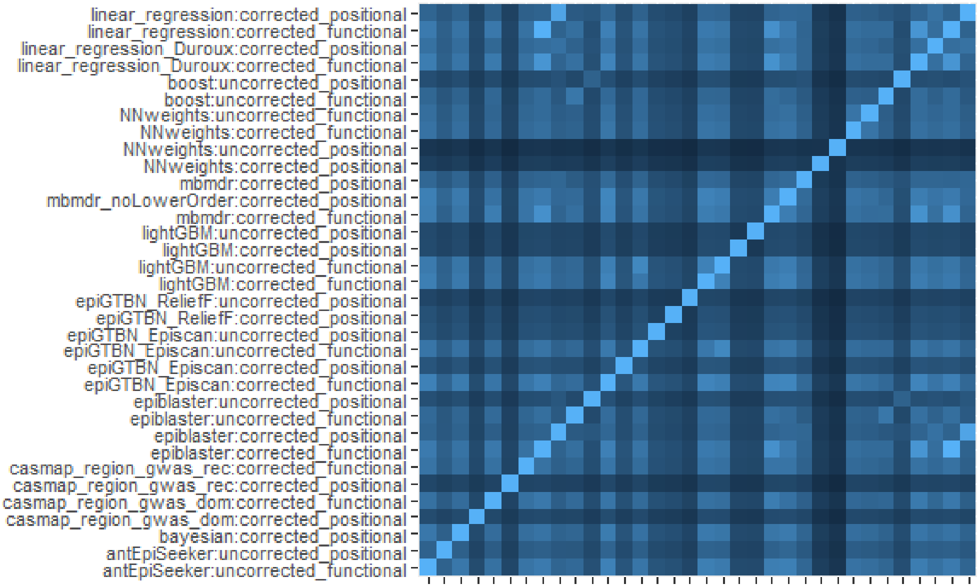

8 Graphical annotation details of epistasis findings

Here, we present GeneMANIA [18] physical interactions connecting NOD2 and TLR5 (Figure S3), and connecting SLC22A4 and SLC22A5 (Figure S4), which were highlighted in Subsection 3.3. We used the GeneMANIA application version 3.6.0, with the reported last database update on 13 August 2021.

9 Simulation results

To further investigate the heterogeneity across methods, we examined the structure and implications of the observed concordance patterns. Tools based on similar statistical assumptions—such as logistic regression–based PLINK-LR and the exhaustive pairwise search implemented by Epi-Blaster— shared a higher proportion of predicted interactions, which was reflected both in the Jaccard index (Fig. SS5) and in their highly similar network topologies. This suggests that these methods may be sensitive to similar types of epistatic effects and signal architectures.

In contrast, MBMDR without codominant adjustment and LightGBM systematically deviated from the remaining tools. For MBMDR without codominant adjustment, this behavior is consistent with its design, which emphasizes higher-order and model-free interaction patterns. LightGBM, as a gradient-boosting framework, can capture complex nonlinear relationships that traditional pairwise tests are inherently unable to model. The clustering of these two methods in the NetANOVA dendrogram reflects their shared tendency to depart from the classical epistasis-detection paradigm.

We also performed an extended evaluation of ensemble aggregation. The union of tools produced 62 distinct edges, demonstrating how dispersed the predictions were across methods. Although the union included 6 true interactions (10%), this fraction remained low relative to the total number of predictions, indicating that no single tool contributed a dominant set of true signals. The complete intersection of tools produced no shared edges, reinforcing the observation that the tools operate largely independently in terms of their top-ranked predictions.

The LCA aggregation reduced the network to 38 edges but still included only 2 true interactions (5%). This outcome illustrates an important methodological point: aggregation is only effective when individual tools show sufficient overlap to form a coherent consensus. In scenarios where tools capture fundamentally different aspects of the interaction structure—as observed here—aggregation may instead dilute signals rather than strengthen them.

Together, these analyses underline that the limited concordance across tools is not merely a consequence of ranking differences but reflects deeper methodological divergence. Incorporating a broader range of tools, including those optimized for rare variants, higher-order interactions, or phenotype-adjusted models, may improve consensus-building in future studies.

10 Higher-order (≥ 3 locus) interactions

While our main analysis focuses on pairwise gene interactions, higher-order epistasis is increasingly recognized as important for complex traits. Modeling interactions among three or more loci poses unique challenges due to the combinatorial expansion of the search space, increased multiple-testing burdens, and sparsity of multilocus genotype cells. These issues are further compounded by linkage disequilibrium (LD) confounding, population structure, missing data, and the difficulty of interpreting multilocus interactions. Here, we outline methodological approaches for higher-order epistasis and describe how our workflow can accommodate them.

Methods and challenges. Several families of approaches explicitly target higher-order epistasis.

1. **Multifactor Dimensionality Reduction (MDR) and derivatives** (GMDR, MB-MDR) collapse multilocus genotype cells to optimize classification and have been widely used and reviewed. MB-MDR additionally incorporates model-based testing, improving error control for quantitative traits [19].

2. **Bayesian frameworks** (e.g., BEAM) model joint architectures and can capture multi-locus effects, trading computational speed for flexibility [20].

3. **Heuristic or exhaustive accelerators** (e.g., AntEpiSeeker; BitEpi for exhaustive 3- and 4-SNP scans with bitwise entropy statistics) reduce runtime while maintaining statistical power [9].

Recent comparisons highlight variable recovery of true three-locus interactions, reinforcing the need for heterogeneity-aware consolidation of results [21].

Representing higher-order results within our framework. Our workflow can accommodate base callers that output tuples at the SNP or gene level. The simplest extension maps variants to genes, projecting each k-tuple into a clique among its member genes, with edge weights reflecting the number or strength of higher-order tuples in which a pair co-occurs. Order-aware weighting can up- or down-weight evidence from 2-way versus 3/4-way interactions. Tools such as ViSEN provide a precedent for visualizing up to three-way interactions on networks [22].

Beyond pairwise projections, two generalisations are possible:

1. **Hypergraph or simplicial-complex SENs**, where each higher-order interaction forms a hyperedge, and network measures such as community detection, centrality, and clustering are computed in the higher-order space [23].

2. **Multilayer SENs**, with separate layers for order-2, order-3, etc., aggregated via order-specific weights prior to netANOVA or LCA.

These designs are fully modular within our pipeline: the aggregation and clustering stages remain unchanged, while SEN construction can replace pairwise edges with (i) clique-expanded edges with order-aware weights or (ii) hyperedges. Implementation of these extensions is ongoing in our group.

11 Recommendations based on application to IBD

Several recommendations come out of this comparison. We observe that different analytic tools produce heterogeneous results. We grouped the methods based on the result's similarity, and we suggest applying at least one analysis per group. We stressed that the modelling framework and search space reduction are two important distinguishing factors between GWAIS analytics. From

the resemblance network (Fig. 2) and the netANOVA clustering (Fig. 3), we recommend applying one method among the linear regression, Epiblaster, MB-MDR and BOOST. The other tools can be chosen depending on the context. For instance, CASMAP looks for large sets of interacting SNP. This method may be more suitable when the goal is to identify regions where epistasis may occur. Also, the Bayesian approach directly outputs results at the gene level and can be applied when no additional choices from the user to go from SNP to gene level is preferred. Epi-GTBN requires prior filtration for computational reasons and is favoured with smaller datasets or in association with filters. We also recommend obtaining gene-level SENs. Indeed, while SNP pairs hardly reproduce in different cohorts, results at the gene level are more likely to be replicable. Aggregating SNP-level results into gene-level epistasis is challenging but allows the inclusion of relevant information from biological interaction databases. Similarly, we suggest applying multiple SNP to gene mapping strategies. We saw that *positional* and *functional* produced different results. We advise applying both approaches to get a complete picture of the biological mechanisms involved in the disease investigated. In particular, we highly recommend including one *functional* mapping as we observed that this increased interpretability. Finally, we demonstrated that aggregative methods could help provide one unique message across multiple homogeneous SENs.

References

[1] Van Lishout, F., Gadaleta, F., Moore, J. H., Wehenkel, L., and Van Steen, K. (2015) gammaMAXT: a fast multiple-testing correction algorithm. *BioData Mining,* **8**(1), 1–15.

[2] Llinares-López, F., Papaxanthos, L., Roqueiro, D., Bodenham, D., and Borgwardt, K. (2019) CASMAP: detection of statistically significant combinations of SNPs in association mapping. *Bioinformatics,* **35**(15), 2680–2682.

[3] Purcell, S., Neale, B., Todd-Brown, K., Thomas, L., Ferreira, M. A., Bender, D., Maller, J., Sklar, P., De Bakker, P. I., Daly, M. J., et al. (2007) PLINK: a tool set for whole-genome association and population-based linkage analyses. *The American journal of human genetics,* **81**(3), 559–575.

[4] Ke, G., Meng, Q., Finley, T., Wang, T., Chen, W., Ma, W., Ye, Q., and Liu, T.-Y. (2017) Lightgbm: A highly efficient gradient boosting decision tree. *Advances in neural information processing systems,* **30**.

[5] Kam-Thong, T., Czamara, D., Tsuda, K., Borgwardt, K., Lewis, C. M., Erhardt-Lehmann, A., Hemmer, B., Rieckmann, P., Daake, M., Weber, F., et al. (2011) EPIBLASTER-fast exhaustive two-locus epistasis detection strategy using graphical processing units. *European Journal of Human Genetics,* **19**(4), 465–471.

[6] Tsang, M., Cheng, D., and Liu, Y. (2017) Detecting statistical interactions from neural network weights. *arXiv preprint arXiv:1705.04977,*.

[7] Walakira, A., Ocira, J., Duroux, D., Fouladi, R., Moškon, M., Rozman, D., and Van Steen, K. (2022) Detecting gene–gene interactions from GWAS using diffusion kernel principal components. *Bmc Bioinformatics,* **23**(1), 1–18.

[8] Guo, Y., Zhong, Z., Yang, C., Hu, J., Jiang, Y., Liang, Z., Gao, H., and Liu, J. (2019) Epi-GTBN: an approach of epistasis mining based on genetic Tabu algorithm and Bayesian network. *BMC bioinformatics,* **20**(1), 1–18.

[9] Wang, Y., Liu, X., Robbins, K., and Rekaya, R. (2010) AntEpiSeeker: detecting epistatic interactions for case-control studies using a two-stage ant colony optimization algorithm. *BMC research notes,* **3**(1), 1–8.

[10] Franzin, A., Sambo, F., and di Camillo, B. (2017) bnstruct: an R package for Bayesian Network structure learning in the presence of missing data. *Bioinformatics,* **33**(8), 1250–1252.

[11] Wan, X., Yang, C., Yang, Q., Xue, H., Fan, X., Tang, N. L., and Yu, W. (2010) BOOST: A fast approach to detecting gene-gene interactions in genome-wide case-control studies. *The American Journal of Human Genetics,* **87**(3), 325–340.

[12] Glover, F., Kelly, J. P., and Laguna, M. (1995) Genetic algorithms and tabu search: hybrids for optimization. *Computers & Operations Research,* **22**(1), 111–134.

[13] Friedman, N., Linial, M., Nachman, I., and Pe'er, D. (2000) Using Bayesian networks to analyze expression data. *Journal of computational biology,* **7**(3-4), 601–620.

[14] Pe'er, D., Regev, A., Elidan, G., and Friedman, N. (2001) Inferring subnetworks from perturbed expression profiles. *Bioinformatics,* **17**(suppl_1), S215–S224.

[15] Friedman, N., Goldszmidt, M., and Wyner, A. (2013) Data analysis with Bayesian networks: A bootstrap approach. *arXiv preprint arXiv:1301.6695,*.

[16] Manduchi, E., Orzechowski, P. R., Ritchie, M. D., and Moore, J. H. (2019) Exploration of a diversity of computational and statistical measures of association for genome-wide genetic studies. *BioData mining,* **12**(1), 1–16.

[17] Koutra, D., Shah, N., Vogelstein, J. T., Gallagher, B., and Faloutsos, C. (2016) Deltacon: Principled massive-graph similarity function with attribution. *ACM Transactions on Knowledge Discovery from Data (TKDD),* **10**(3), 1–43.

[18] Mostafavi, S., Ray, D., Warde-Farley, D., Grouios, C., and Morris, Q. (2008) GeneMANIA: a real-time multiple association network integration algorithm for predicting gene function. *Genome Biology,* **9**, S4.

[19] Ritchie, M. D., Hahn, L. W., Roodi, N., Bailey, L. R., Dupont, W. D., Parl, F. F., and Moore, J. H. (2001) Multifactor-dimensionality reduction reveals high-order interactions among estrogen-metabolism genes in sporadic breast cancer. *American Journal of Human Genetics,* **69**(1), 138–147.

[20] Zhang, Y. and Liu, J. (2007) Bayesian inference of epistatic interactions in case-control studies. *Nature Genetics,* **39**, 1167–1173.

[21] Russ, D., Williams, J. A., Cardoso, V. R., Bravo-Merodio, L., Pendleton, S. C., Aziz, F., Acharjee, A., and Gkoutos, G. V. (2022) Evaluating the detection ability of a range of epistasis detection methods on simulated data for pure and impure epistatic models. *Plos one,* **17**(2), e0263390.

[22] Hu, T., Chen, Y., Kiralis, J. W., and Moore, J. H. (2013) ViSEN: methodology and software for visualization of statistical epistasis networks. *Genetic Epidemiology,* **37**(3), 283–285.

[23] Battiston, F., Cencetti, G., Iacopini, I., Latora, V., Lucas, M., Patania, A., Young, J.-G., and Petri, G. (2020) Networks beyond pairwise interactions: structure and dynamics. *Physics Reports,* **874**, 1–92.

Fig. S2: All SEN networks.

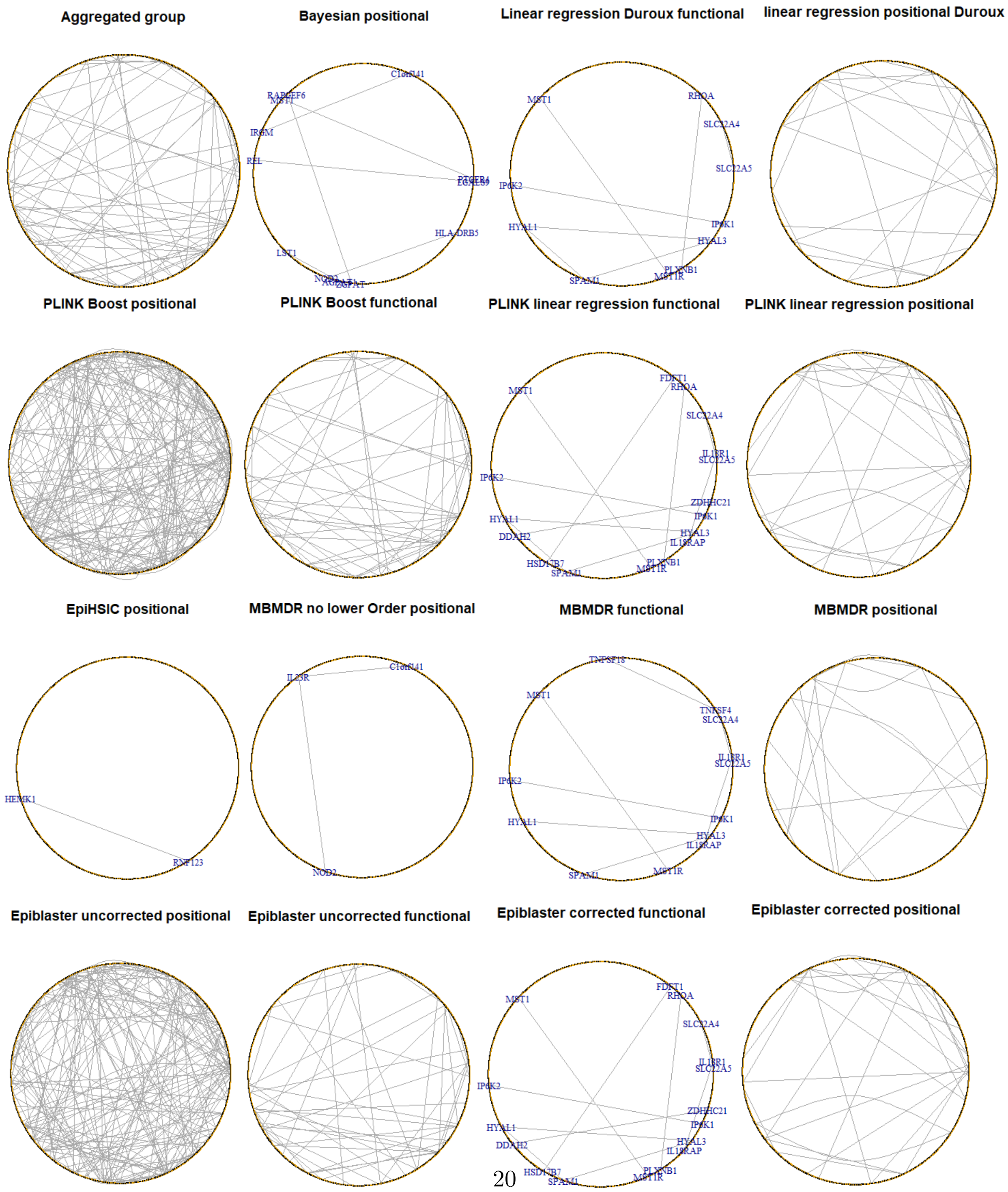

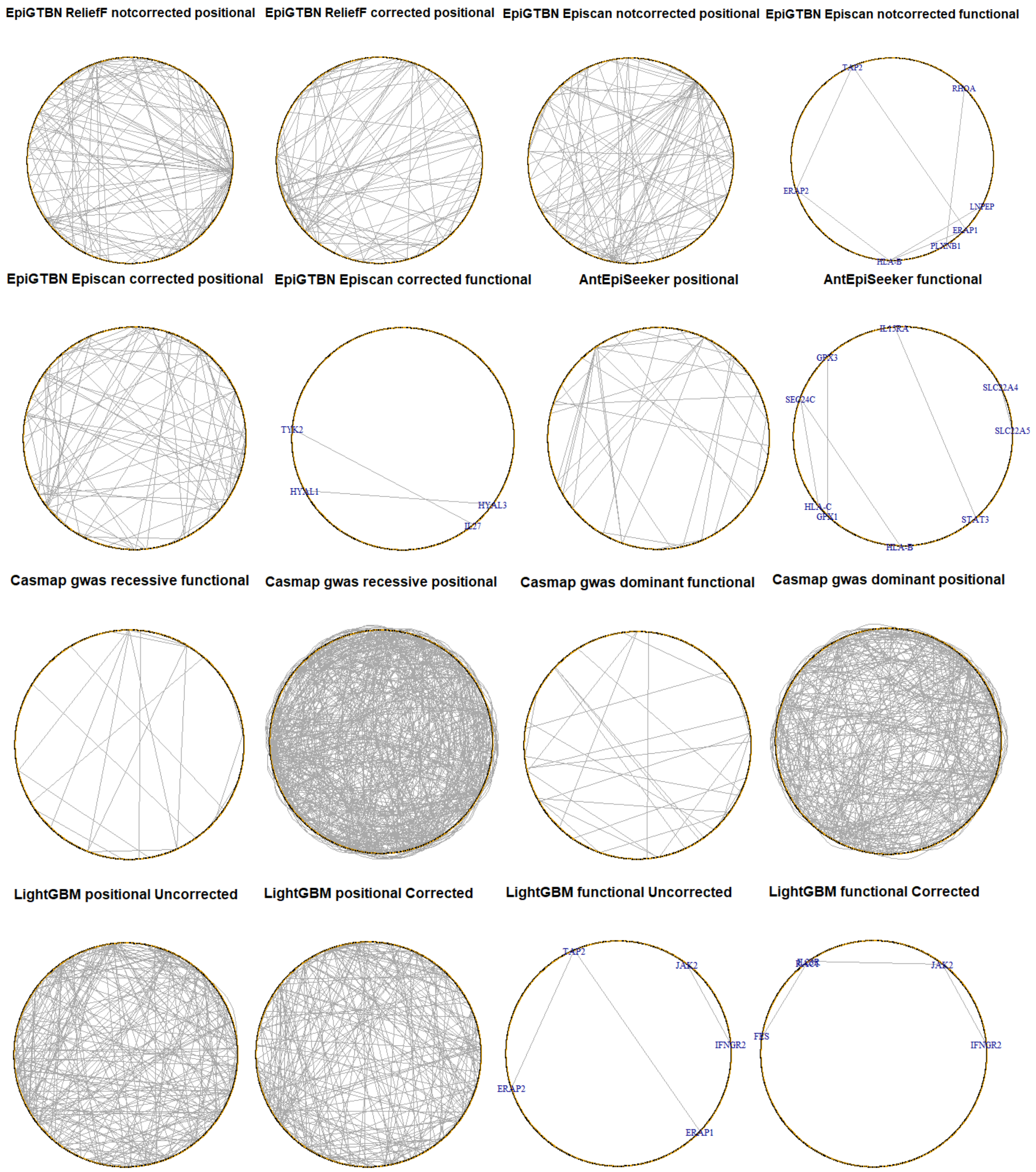
EpiGTBN ReliefF notcorrected positional
EpiGTBN ReliefF corrected positional
EpiGTBN Episcan notcorrected positional
EpiGTBN Episcan notcorrected functional
TAP2
RHOA
ERAP2
LNPEP
ERAP1
PLXNB1
HLA-B
EpiGTBN Episcan corrected positional
EpiGTBN Episcan corrected functional
TYK2
HYAL1
HYAL3
IL27
AntEpiSeeker positional
AntEpiSeeker functional
IL15RA
GPX3
SLC22A4
SEC24C
SLC22A5
HLA-C
GPX1
STAT3
HLA-B
Casmap gwas recessive functional
Casmap gwas recessive positional
Casmap gwas dominant functional
Casmap gwas dominant positional
LightGBM positional Uncorrected
LightGBM positional Corrected
LightGBM functional Uncorrected
TAP2
JAK2
IFNGR2
ERAP2
ERAP1
LightGBM functional Corrected
JAK2
FES
IFNGR2

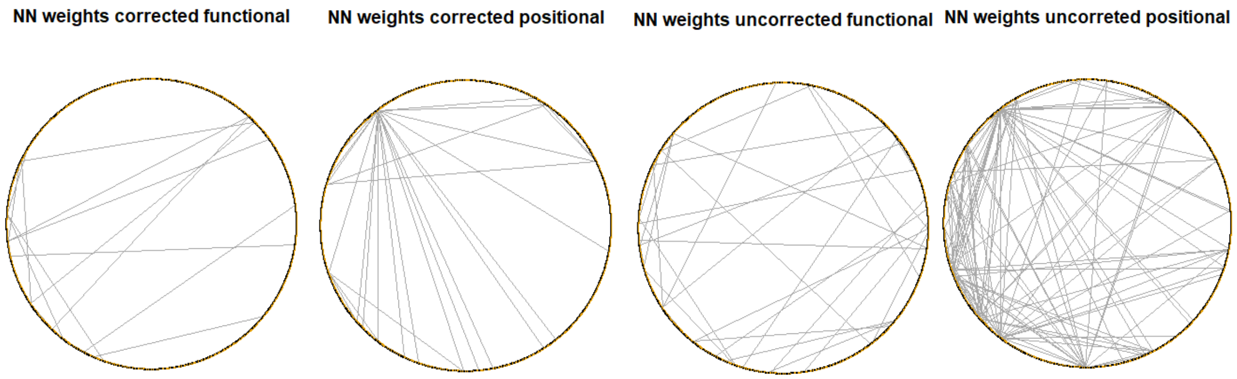

Fig. S3: NOD2 and TLR5 involvement in documented physical interactions.

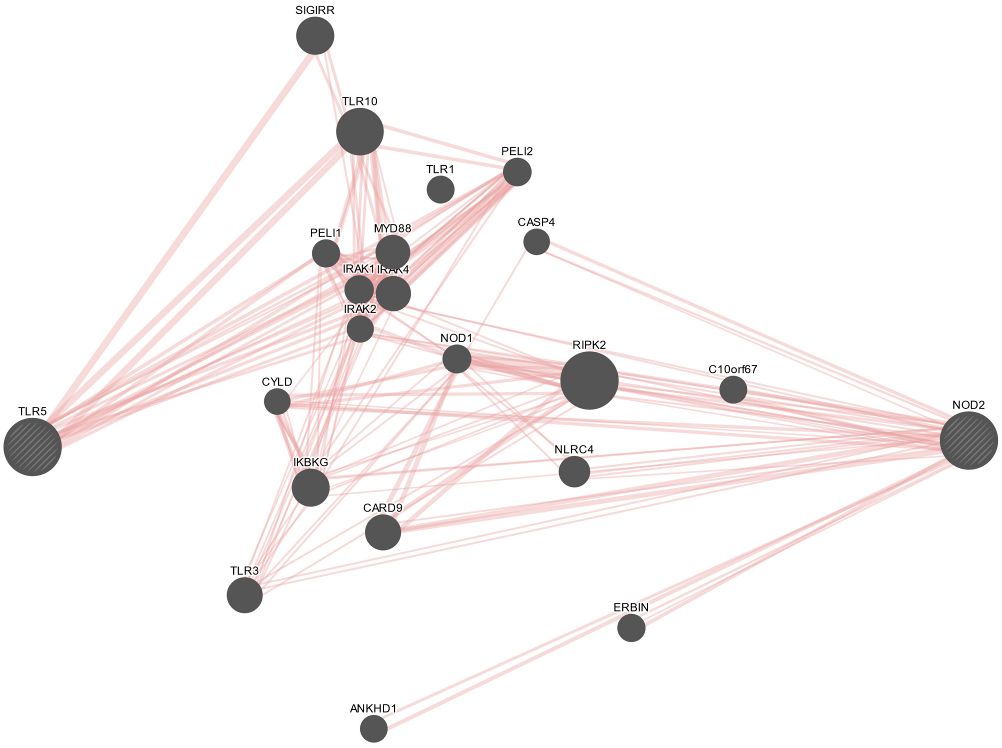

Fig. S4: SLC22A4 and SLC22A5 involvement in documented physical interactions.

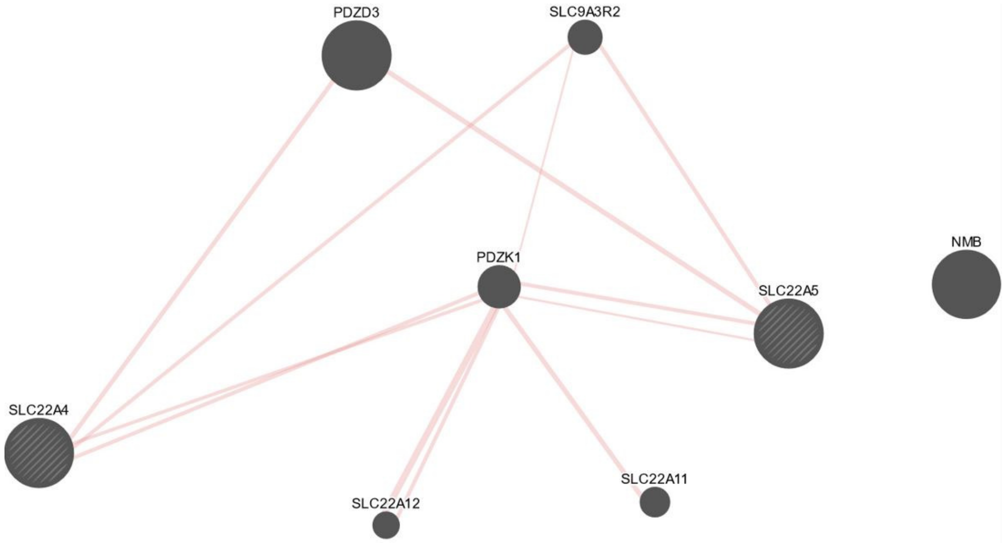

Fig. S5: Similarity of epistasis tools by overlap of top 15 pairs.

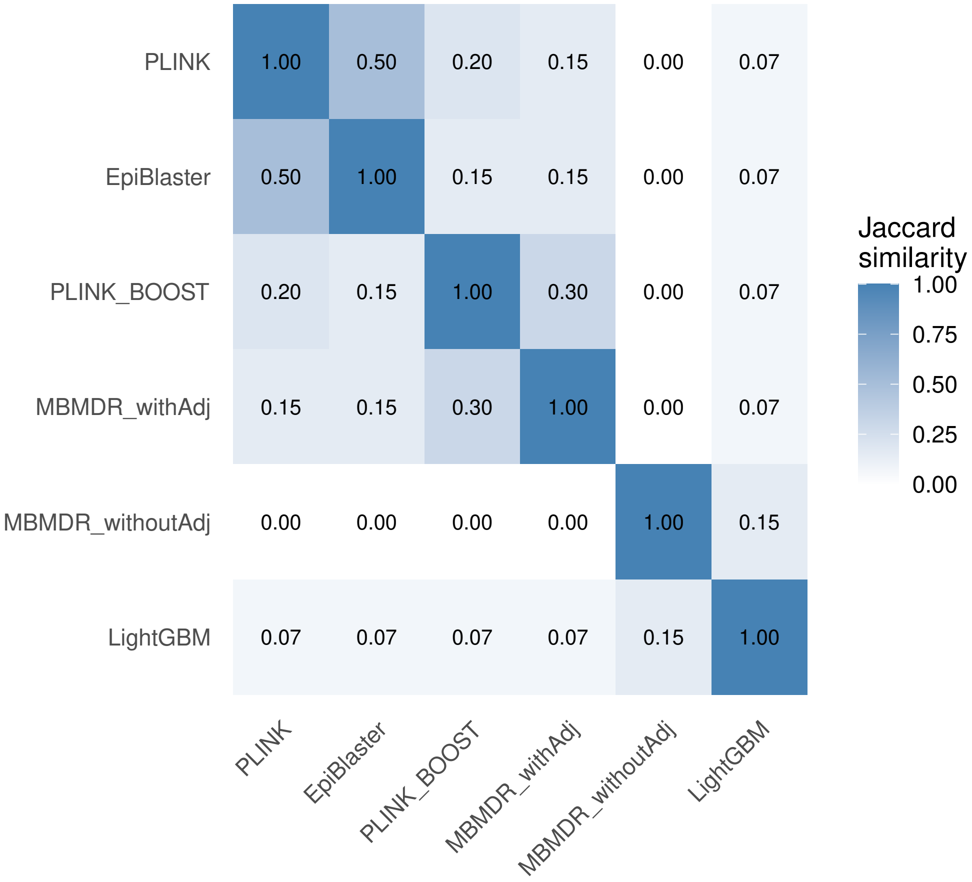

Fig. S6: NetANOVA dendrogram cluster epistasis detection tools based on their similarity structure.

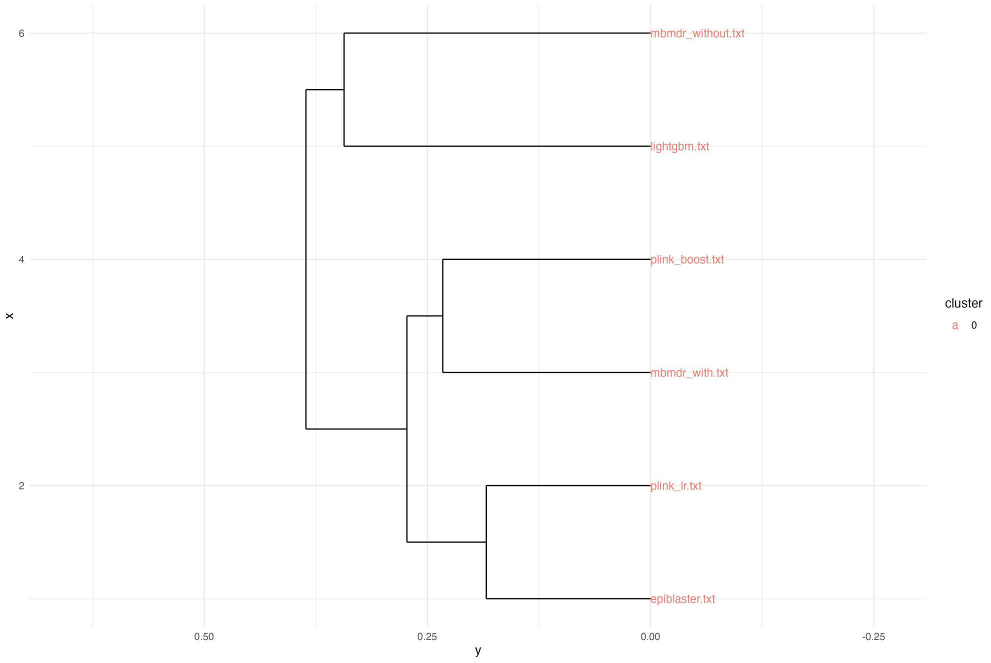

Supplement: Suppl_Turning_heterogeneity_of_statistical_epistasis_networks_to_advantage_bbaf699 [file suppl_turning_heterogeneity_of_statistical_epistasis_networks_to_advantage_bbaf699.pdf]
